# Nonadiabatic Charge Transfer within Photoexcited Nickel Porphyrins

**DOI:** 10.1021/acs.jpclett.4c00375

**Published:** 2024-03-26

**Authors:** Maria A. Naumova, Gheorghe Paveliuc, Mykola Biednov, Katharina Kubicek, Aleksandr Kalinko, Jie Meng, Mingli Liang, Ahibur Rahaman, Mohamed Abdellah, Stefano Checchia, Frederico Alves Lima, Peter Zalden, Wojciech Gawelda, Christian Bressler, Huifang Geng, Weihua Lin, Yan Liu, Qian Zhao, Qinying Pan, Marufa Akter, Qingyu Kong, Marius Retegan, David J. Gosztola, Mátyás Pápai, Dmitry Khakhulin, Max Lawson Daku, Kaibo Zheng, Sophie E. Canton

**Affiliations:** † Deutsches Elektronen-Synchrotron DESY, Notkestr. 85, 22607 Hamburg, Germany; ‡ Département de Chimie Physique, Université de Genève, Quai E. Ansermet 30, CH-1211 Genève, Switzerland; § European XFEL, Holzkoppel 4, 22869 Schenefeld, Germany; ∥ The Hamburg Centre for Ultrafast Imaging, University of Hamburg, Luruper Chaussee 149, 22761 Hamburg, Germany; ⊥ Fachbereich Physik, Universität Hamburg, Notkestraße 9-11, 22607 Hamburg, Germany; # Department of Chemistry, Technical University of Denmark, Kongens Lyngby DK-2800, Denmark; ¶ Chemical Physics and NanoLund, Lund University, SE-221 00 Lund, Sweden; ■ Department of Chemistry, United Arab Emirates University, P.O. Box 15551, Al Ain, United Arab Emirates; ● Department of Chemistry, Qena Faculty of Science, South Valley University, Qena 83523, Egypt; ▲ ESRF - The European Synchrotron, 71, avenue des Martyrs, CS 40220, 38043 Grenoble Cedex 9, France; ▼ Departamento de Química, Universidad Autónoma de Madrid, Madrid 28049, Spain; ⬢ IMDEA-Nanociencia, Calle Faraday 9, Madrid 28049, Spain; □ Faculty of Physics, Adam Mickiewicz University, Poznan 61-614, Poland; ○ Department of Physics, Yantai University, 30 Qingquan Road, Yantai 264005, China; △ Synchrotron Soleil, L’Orme des Merisiers, 91190 Saint-Aubin, France; ▽ Center for Nanoscale Materials, Argonne National Laboratory, 9700 South Cass Avenue, Lemont, Illinois 60439, United States; ⬡ HUN-REN Wigner Research Center for Physics, P.O. Box 49, Budapest H-1525, Hungary

## Abstract

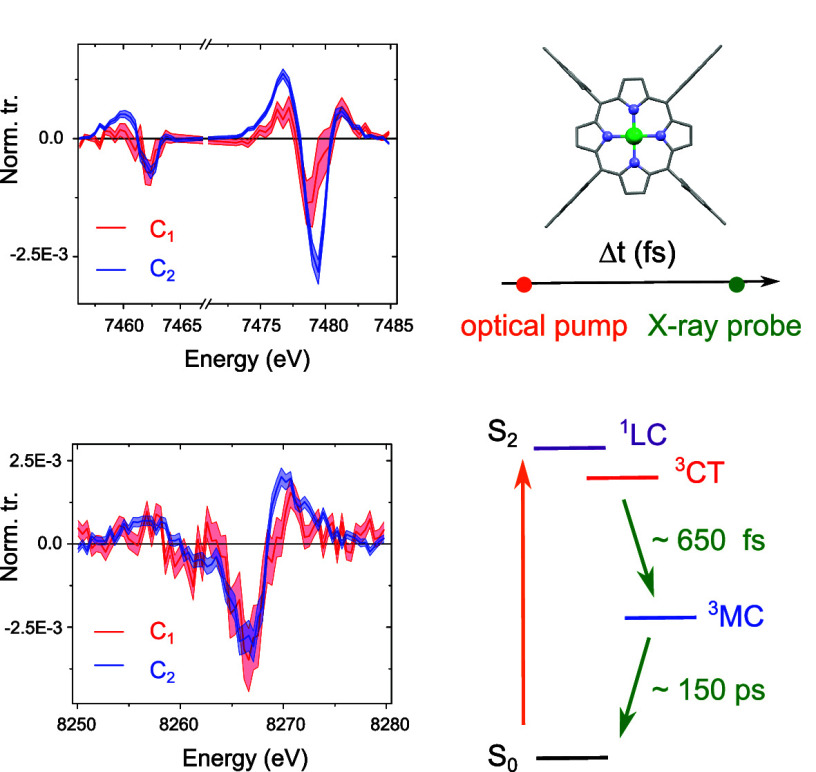

Metalloporphyrins with open d-shell ions can drive biochemical energy cycles. However, their utilization in photoconversion is hampered by rapid deactivation. Mapping the relaxation pathways is essential for elaborating strategies that can favorably alter the charge dynamics through chemical design and photoexcitation conditions. Here, we combine transient optical absorption spectroscopy and transient X-ray emission spectroscopy with femtosecond resolution to probe directly the coupled electronic and spin dynamics within a photoexcited nickel porphyrin in solution. Measurements and calculations reveal that a state with charge-transfer character mediates the formation of the thermalized excited state, thereby advancing the description of the photocycle for this important representative molecule. More generally, establishing that intramolecular charge-transfer steps play a role in the photoinduced dynamics of metalloporphyrins with open d-shell sets a conceptual ground for their development as building blocks capable of boosting nonadiabatic photoconversion in functional architectures through “hot” charge transfer down to the attosecond time scale.

Metalloporphyrins (MPs) incorporating 3d transition metal ions with closed or open d-shells are primary reactants in biochemical energy cycles.^1−5^ Building upon the first reports of porphyrin π-ring synthesis in the 1930s,^6,7^ modern protocols have expanded the MP family to encompass most of the stable elements.^8−13^ The so-called “MP periodic table”^14,15^ rationalizes how the nature of the metal ion imparts unique photophysical properties, placing MPs at the focus of a cross-disciplinary research effort that targets the bioinspired production, conversion, transduction and storage of renewable energy.^16−20^ In contrast, the integration of MPs containing transition metal ions with an open 3d-shell in photoconversion applications remains limited despite strong prospects associated with their coupled electronic and magnetic activities. This status can be traced back to the fact that, although the initial photoexcitation is delocalized over the porphyrin π-ring as for their closed-shell congeners, the pathways of intramolecular relaxation are rapidly obscured by the participation of intermediate states that involve the d-levels of the metal ions.^16−18^ Several decades of investigations with electronic spectroscopy have shown that it is rarely possible to unambiguously differentiate (d,d), (π,d), and (d,π) states in the UV–visible and near-infrared spectral ranges.^21−23^ Ultrafast techniques with inherent structural sensitivity such as time-resolved Raman spectroscopy (tr-RSS)^24−26^ and, recently, time-resolved X-ray absorption spectroscopy (tr-XAS) on the picosecond (ps) and femtosecond (fs) time scales^23,27−32^ are partially challenged by the lack of direct spin-sensitivity so that they tend to return highly correlated rate constants. As a result, for most open d-shell MPs, the description of the photocycle is not exhaustive, and the photoredox capabilities of the excited states cannot be fully explored. Here, we combine transient optical absorption spectroscopy (tr-OAS) with transient X-ray emission spectroscopy (tr-XES), both achieving femtosecond resolution, in order to track directly the coupled electronic and spin dynamics within a photoexcited nickel (Ni) porphyrin in solution. The measurements reveal a distinct intermediate state with charge-transfer (CT) character in the excited triplet manifold. Supported by time-dependent density functional theory (TD-DFT) and excited-state DFT (e-DFT) theoretical calculations, the observations allow proposing a pathway connecting the singlet Franck–Condon (FC) state with excitation localized on the π-ring (i.e., ligand-centered (LC) character) to the triplet metastable (d,d) state (i.e., metal-centered (MC) character). Besides completing the description of the photocycle for this important representative molecule, this study also outlines novel directions for utilizing photoexcited Ni porphyrins and other open d-shell MPs to trigger “hot” charge-transfer events in large functional architectures down to the attosecond time scale.

The basic Ni porphyrin molecule is formed through metalation of the porphin dianion (P^2–^) by Ni(II) cations. The Ni(II) tetrakis(2,4,6-trimethylphenyl)porphyrin (NiTMP) possesses aryl substituents added at the four meso positions. The chemical structure is shown in Figure 1a.

**Figure 1:**
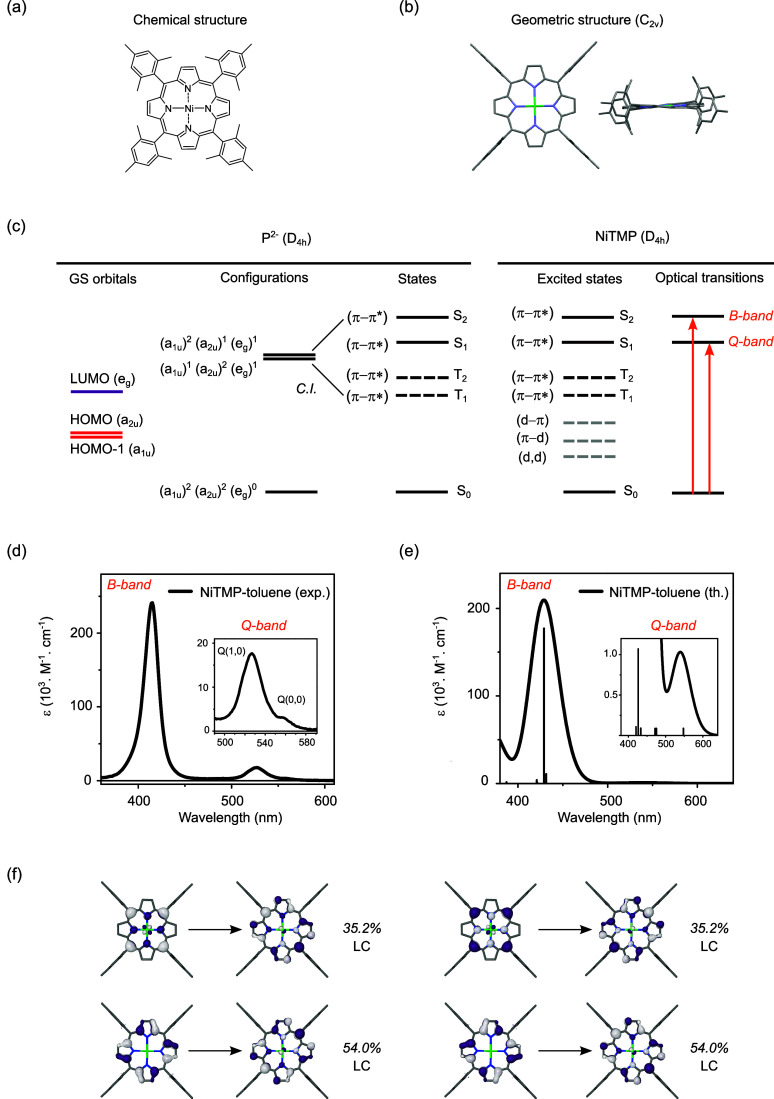
(a) Chemical structure of NiTMP. (b) Geometric structure for the ground state of NiTMP in toluene obtained using DFT optimizations in C_2v_ symmetry with the PBE functional. (c) The Gouterman model and the associated molecular states for P^2–^ and NiTMP. The occupied a_2u_ HOMO and a_1u_ HOMO−1 are shown in red; the unoccupied LUMOs e_g_ are shown in purple. The ligand-centered (LC) states of singlet multiplicity are indicated as solid black lines, while the LC state of triplet multiplicity are indicated as dashed black lines. The states of ligand-to-metal charge-transfer (LMCT) character, metal-to-ligand charge-transfer (MLCT) character, and metal-center (MC) character are indicated as dashed gray lines. The (π,π*) transitions centered on the ligand π-ring dominate the absorption spectrum in the UV–visible. They are indicated by the orange arrows. (d) Experimental absorption spectrum of NiTMP in toluene. The inset zooms on the Q-band. (e) Theoretical absorption spectrum of NiTMP in toluene (using TD-DFT with the PBE functional). (f) Dominant occupied NTO → virtual NTO transitions depicting the main electronic transitions underlying the Soret band, along with their weights.

The experimental details for the synthesis of the molecule are given in S.I.1. The structural optimization of NiTMP with density functional theory (DFT) calculations performed in C_2v_ symmetry delivers the geometry of the 1 ^1^A_1_ singlet ground state in toluene (Figure 1b). The computational details are given in S.I.2 (S1). The value of the average Ni–N bond length, noted as *R*, is 1.953 Å. The molecule departs from planarity, as the ring presents clear ruffling. This type of deformation is ascribed to the fact that the Ni–N bonds are shorter than the radius of the N_4_ cavity in the planar π-ring of free P^2–^.^33^ The Ni(II) ion is in the (d_*xy*_)^2^(d_*xz*_)^2^(d_*yz*_)^2^(d_*z*_^2^)^2^ (*S* = 0) low-spin (LS) electronic configuration. The Kohn–Sham orbitals are displayed in S.I.2 (S2).

The optical spectra of closed d-shells MPs are very well described by the so-called Gouterman model involving four orbitals, namely the two nearly degenerate a_2u_ HOMO, a_1u_ HOMO–1^34^ (red) and the doubly degenerate e_g_ LUMO (purple) of P^2–^ (Figure 1c).^35,36^ Within this model, pronounced configuration interaction (C.I.) between the excited states originating from the (a_1u_)^2^(a_2u_)^1^(e_g_)^1^ and (a_1u_)^1^(a_2u_)^2^(e_g_)^1^ configurations produces two (π,π*) singlet states, S_1_ and S_2_, and two (π,π*) triplet states, T_1_ and T_2_, for which the excitation is localized on the π-ring, i.e., with ligand-centered (LC) character. These states are indicated as solid and dashed black lines in Figure 1c. The d-electrons of the Ni(II) ion perturb these energetics only slightly, with possible formation of (π,d)/(d,π) states of charge-transfer (CT) character and (d,d) states of metal-centered (MC) character within the singlet and triplet manifolds. These states are indicated as dashed gray lines. The two optically allowed transitions, S_0_ → S_2_ and S_0_ → S_1_, yield the strong Soret B-band and the weaker Q-band (orange arrows). Direct transitions to the triplet states are optically forbidden in the absence of spin–orbit coupling (SOC).

**Figure 2:**
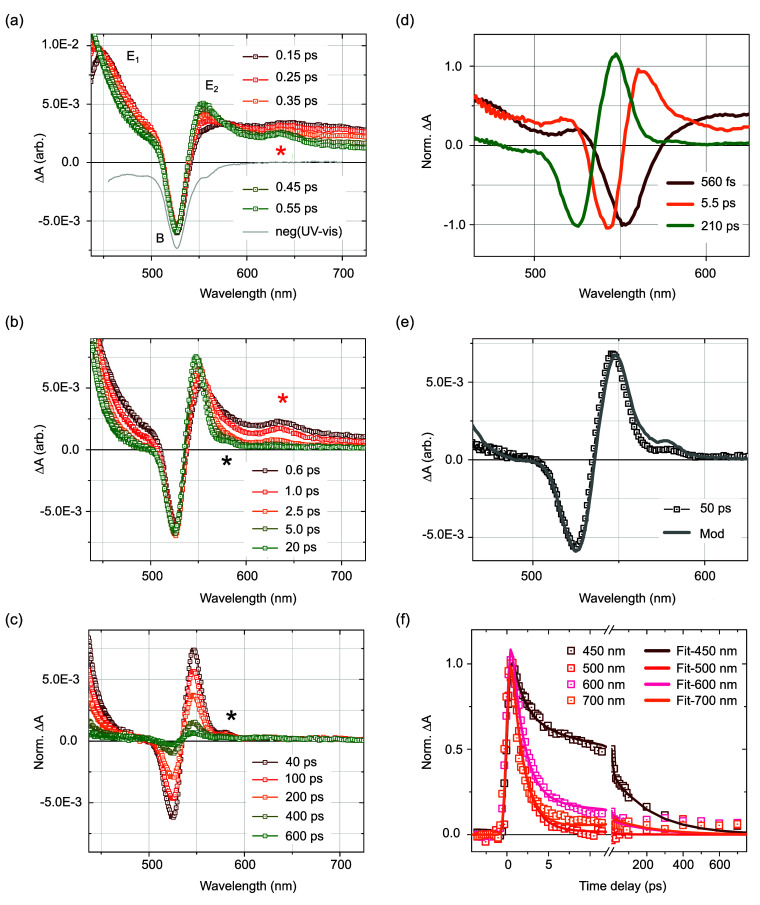
Transient optical absorption spectra for given time delays Δ*t* across the (a) subpicosecond, (b) tens of picosecond, and (c) subnanosecond temporal windows. The negative of the UV–vis absorbance is also displayed after scaling in panel a (gray line). (d) Three components extracted from a global-fit analysis using a sequential model based on three time constants. (e) Transient optical absorption spectrum observed for a time delay Δ*t* of 50 ps (open squares) and the model trace (solid line) (see main text). (f) Kinetics at 450, 500, 600, and 700 nm and their fits extracted from the global-fit analysis (see main text).

The UV−vis absorption spectrum of NiTMP in toluene is shown in Figure 1d. The B-band is centered at around 415 nm. The Q-band displays a vibronic substructure, with Q(1,0) and Q(0,0) at 527 and 558 nm (inset of Figure 1d). The experimental spectrum is well reproduced by time-dependent DFT (TD-DFT) calculations using the PBE functional (Figure 1e). The main transitions between the occupied and virtual natural transition orbitals (NTOs) underlying the Soret band are depicted in Figure 1f. They are located on the porphyrin ring and do not implicate any electronic density at the Ni(II) center, as expected from a (π,π*) transition (S3 and S4 in S.I.2).

The ultrafast photoinduced dynamics of NiTMP were first monitored with femtosecond tr-OAS in the UV–visible range. The setup is described in S.I.3. NiTMP was dissolved in toluene and excited in the B-band at 400 nm. Transient optical absorption spectra acquired over the subpicosecond, tens of picosecond, and hundreds of picosecond temporal windows are presented in Figure 2a–c for specific pump–probe delays Δ*t*. The incident fluence was set in the linear regime of photoexcitation (S.I.3).

In Figure 2a, the GS bleach (GSB) of the Q(1,0) band at 527 nm (feature B) appears within the instrument response function (IRF) defined by a Gaussian function of full-width half-maximum (fwhm) ∼ 120 fs, along with positive excited-state absorption (ESA) signals on the blue side (feature E_1_) and the red side (feature E_2_) of feature B. A small peak forms around 635 nm (indicated by a red star). Isosbestic points are observed at 444 and 577 nm. The inverse of the GS absorbance is shown as a gray trace. In Figure 2b, the intensity of E_1_ decays rapidly, while E_2_ undergoes profound alterations as it narrows and blue-shifts with the small peak localizing toward shorter wavelengths (indicated by the black star). In Figure 2c, the transient signal of the lowest excited state exhibits a derivative-like profile with a shoulder at 580 nm (indicated by the black star). It decays on the ∼200 ps time scale with a clear isosbestic point at 534 nm. Early studies have assigned this metastable state as a (d_*z*^2^_, d_*x*^2^–*y*^2^_) state with MC character owing to the derivative-like profile. This distinctive spectral line shape was proposed to result from the superposition of the GSB signal with an ESA signal corresponding to the same GS spectrum but shifted to lower energy. The spectral red-shift is attributed to the repulsion between the electrons populating the a_2u_ ring-based orbital and the d_*x*^2^–*y*^2^_ metal-based orbital that both place electronic density in the vicinity of the N atoms. Such interaction destabilizes the a_2u_ HOMO, while leaving the e_g_ LUMO unaffected, thereby only reducing the HOMO–LUMO gap (see Figure 1c).^37,38^ Although the spin multiplicity of the (d,d) state cannot be inferred from the optical measurement, it is conventionally assumed to become triplet through intersystem crossing (ISC), in line with Hund’s rule and the absence of detectable stimulated emission in Figure 2a. The individual kinetics are expected to be probe-dependent, considering the nonadiabatic nature of the dynamics following photoexcitation. Nevertheless, a global-fit analysis based on a simple sequential kinetic model including three exponential functions captures the essential intensity redistribution over extended ranges of wavelengths and times. The three components are displayed in Figure 2d, after scaling their minima to −1 for visualization purposes. The best-fit values of the time constants are 560 ± 60 fs (red line), 6 ± 1 ps (orange line), and 210 ± 20 ps (green line). Figure 2e shows the transient optical absorption spectrum observed at 50 ps (dotted line). The model trace (solid line) is constructed as the difference between the ground-state UV–visible spectrum red-shifted by ∼20 nm and the ground-state UV–visible spectrum itself. The excellent agreement between the traces supports the assignment of the metastable state as a (d,d) state with MC character. Selected kinetics at 450, 500, 600, and 700 nm are presented in Figure 2f, along with their corresponding best-fit traces. The spectral dynamics over the first several tens of ps reflect the formation of the (d,d) state through competing internal conversion (IC), intersystem crossing (ISC), and intramolecular vibrational energy redistribution (IVR).^26,39^ As noted above, the absence of stimulated emission in the tr-OAS measurements indirectly suggests that the excited-state manifold acquires a triplet spin-multiplicity on the subhundred fs time scale through ISC. However, the multiexponential evolution of the kinetics does not display clear-cut sensitivity toward the extent of the excitation localization at the Ni center in the metastable MC state.

Therefore, femtosecond tr-XES measurements were carried out at the FXE Instrument of the European XFEL facility^40−43^ in order to track directly the dynamics of the spin-multiplicity at the Ni(II) center during the relaxation across the excited-state manifold. The setup is shown in S11 of S.I.4. The data acquisition and analysis procedures are described in S.I.4. In the hard X-ray regime, the radiative relaxation upon 1s core-ionization produces the emission lines 2p_1/2,3/2_ → 1s (Kα_1,2_) and 3p_1/2,3/2_ → 1s (Kβ). They are sensitive to the coupled changes in spin multiplicity and electronic structure.^44^Figure 3a,b shows the Kα_1,2_ and Kβ spectra for NiTMP in toluene in its GS. Figure 3c,d presents the difference traces [(laser_on) – (laser_off)] delivering the Kα_1,2_ and Kβ tr-XES spectra following photoexcitation at 400 nm with a fluence of ∼4 mJ/cm^2^ (linear regime) for the optical pump-X-ray probe time delays Δ*t* of 1 and 50 ps (S12 in S.I.5 and S13, S14 in S.I.6). The similarity between the two transient XES spectra demonstrates that the MC character is acquired on the subpicosecond time scale. Taking the summed absolute value of the integrated areas under the transient difference signals as figure of merit (FOM) and tracking it as a function of pump–probe time delay allows accessing the kinetics of the relaxation with spin sensitivity.^44,45^Figure 3e,f shows the Kα_1,2_ and Kβ kinetics, along with their single-exponential decay fits (black solid and dotted lines) where the IRF fwhm is set to 110 fs, delivering lifetimes of ∼161 ± 10 and ∼168 ± 11 ps for the metastable state. The nominal powers and the best-fit values for the parameters are S16, S17 and S18 in S.I.7. The values of the single-exponential time constants are similar for the optical and X-ray spectroscopic measurements, ascertaining that both techniques are probing the decay of the metastable state.

**Figure 3:**
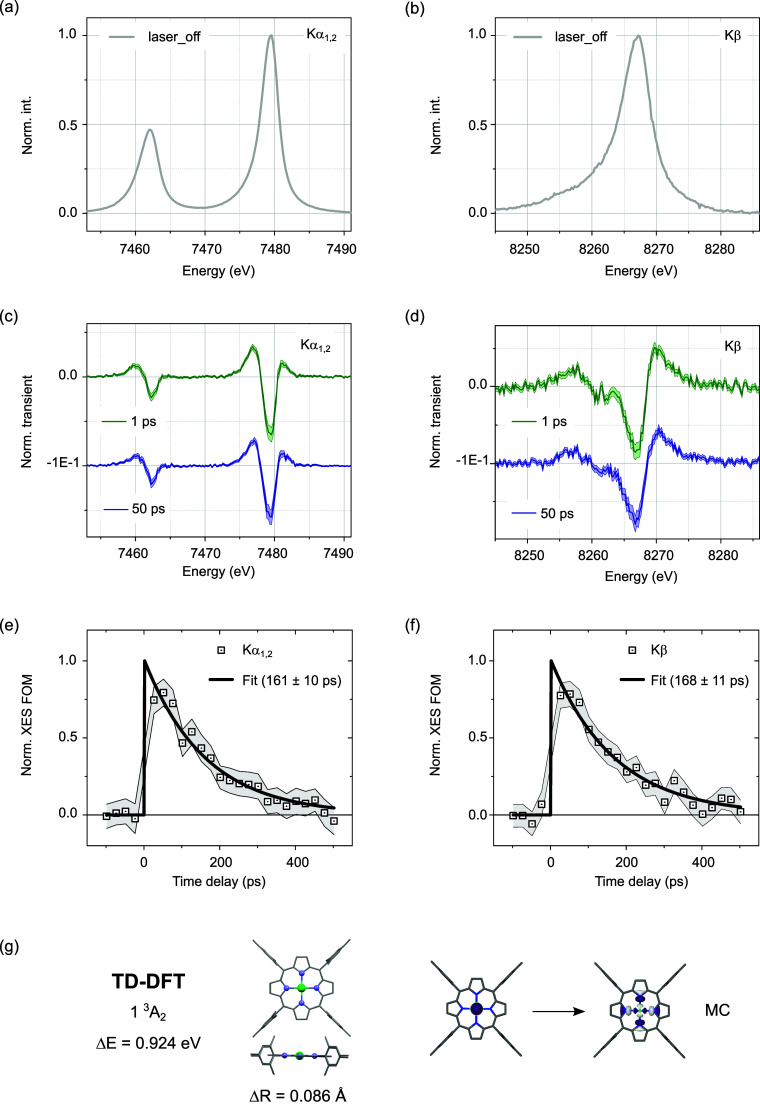
X-ray emission spectroscopy measurements for NiTMP in toluene. (a) Kα_1,2_ spectrum and (b) Kβ spectrum for the ground state of NiTMP toluene. (c) Transient difference Kα_1,2_ and (d) Kβ tr-XES spectra for pump–probe delays Δ*t* of 1 ps (green) and 50 ps (blue). The shaded areas around the lines indicate the experimental error bars (rms noise). The excitation wavelength is 400 nm. (e) Normalized Kα_1,2_ and (f) Kβ kinetics of the XES FOM on the subnanosecond time scale with their single-exponential decay fit (solid and dotted lines, see main text). (g) Relaxed TD-DFT structure of the lowest triplet state 1 ^3^A_2_. The adiabatic energy difference Δ*E* and the average bond length elongation Δ*R* compared to the singlet ground state is Δ*R* = 0.086 Å.

The characteristics of the metastable state are established with theoretical methods. The lowest triplet states obtained from DFT and TD-DFT applying the C_2v_ constraint are of ^3^A_2_ symmetry. The optimized DFT/relaxed TD-DFT structure of this state denoted 1 ^3^A_2_ exhibits an average Ni–N bond elongation Δ*R* of 0.072/0.086 Å with concurrent planarization of the porphyrin π-ring. The molecular geometries are characterized respectively in S5, S7, S8, and S9 of S.I.2 and Figure 3g. Examining the Kohn–Sham orbitals from DFT for the 1 ^1^A_1_ ground state and 1 ^3^A_2_ shown in S.I.2 (S6) reveals that the two states differ by the promotion of an electron from a d_*z*^2^_-like MO to a d_*x*^2^–*y*^2^_-like MO. Examining the NTOs from TD-DFT associated with the relaxed 1 ^3^A_2_ confirms its formation via an electronic transition from a d_*z*^2^_-like MO to a d_*x*^2^–*y*^2^_-like MO (Figure 3g). Overall, both methods establish that the lowest excited triplet state 1 ^3^A_2_ displays an MC character with an expanded and planar core compared to the 1 ^1^A_1_ ground state correlated to the population of a d_*x*^2^–*y*^2^_-like MO.

The early spin dynamics were followed with tr-XES on the subpicosecond time scale with a fluence of ∼2 mJ/cm^2^ (S12 in S.I.5). Transient XES spectra were acquired on a fine temporal grid over 2 ps. Figure 4a,b displays the transient Kα_1,2_ and Kβ traces averaged around the central pump–probe delays Δ*t* of −50 and 50 fs (binned over a 100 fs time window) as well as 200, 450, and 800 fs (binned respectively over a 200, 300, and 400 fs time window) across the energy range where the transient signal is detected. Figure 4c,d shows the Kα_1,2_ and Kβ kinetics, with their fit as single-exponential rise tracking the formation of the MC state (S23, S27 and S28 of S.I.7).

**Figure 4:**
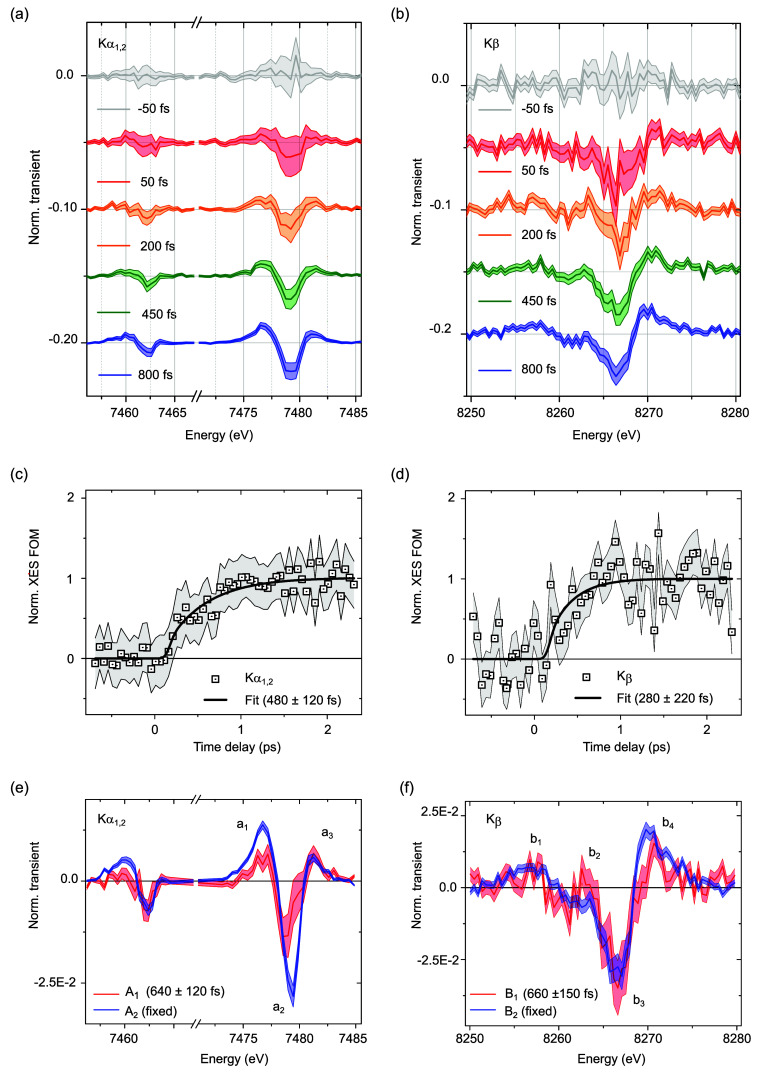
(a) Difference Kα_1,2_ spectra and (b) Kβ spectra for NiTMP in toluene photoexcited at 400 nm for different pump–probe delays. (c) Kα_1,2_ and (d) difference Kβ kinetics (open squares) and their single-exponential fits (solid lines) for the rise (see main text). (e) The A_1_ and A_2_ components and (f) the B_1_ and B_2_ components extracted from global fitting with two-point rebinning (see main text).

However, close inspection of the profiles reveals a departure from uniform growth, with clear alterations in spectral weight as a function of Δ*t*. The differences in feature positions and amplitude ratios flag the participation of an intermediate species in the ultrafast formation of the metastable MC species. Focusing on the first 2 ps, the global-fit analysis of the Kα_1,2_ and Kβ lines based on a single-exponential rise convoluted by a Gaussian IRF of 110 fs fwhm^41^ captures the early dynamical evolution (S.I.7). The fitting model and the best-fit parameters are summarized in S15, S19, S20, S21, S22, S24, S25, S26 of S.I.7. The components A_1_, A_2_ for Kα_1,2_ and B_1_, B_2_ for Kβ are shown in Figure 4e,f with two-point rebinning. The first components (A_1_ for Kα_1,2_ and B_1_ for Kβ, in blue) appear within the IRF of the tr-XES measurement. They evolve toward the second component (A_2_ for Kα_1,2_ and B_2_ for Kβ, in red) with a time-constant τ_1_ ∼ 640 ± 120 fs (Kα_1,2_) and τ′_1_ ∼ 660 ± 150 fs (Kβ). The lineshapes of components A_2_ and B_2_ are very similar to the Kα_1,2_ and Kβ transient difference spectra acquired at a pump–probe delay Δ*t* of 1 and 50 ps displayed in Figure 3c,d so that they can be unambiguously identified as the fingerprints of the triplet state (d,d) of MC character. In contrast, the lineshapes of A_1_ and B_1_ present clear variations. The amplitudes of features a_1_ and a_2_ in the Kα_1_ range of A_1_ are smaller than those of A_2_, while the feature a_3_ is slightly broader. The amplitudes of all transient features in the Kα_2_ range are smaller. The amplitudes of features b_1_, b_3_, and b_4_ in the Kβ range of B_1_ are smaller than those of B_2_, while b_2_ appears suppressed. In other words, the intermediate species exhibits transient XES signatures that are distinguishable from the ones of the triplet MC state displayed in Figure 3c,d. Moreover, it should be noted here that the vertical transition to the singlet FC state (without geometrical changes) does not involve any appreciable electronic density at the metal ion (Figure 1f) so that it could be expected to appear predominantly silent in the tr-XES measurement. Therefore, the intermediate species possesses CT character. The time-dependent concentrations of the CT and MC species are shown in S29 of S.I.7. They are based on the parameters extracted from the global-fit analysis of the Kα_1,2_ lines (i.e., for a formation time of 640 fs).

The assignment of the intermediate CT state is then investigated with theoretical methods. The excited states of triplet spin multiplicity lying above the lowest metastable 1 ^3^A_2_ triplet state and below the initial excitation energy were first characterized with TD-DFT calculations for each symmetry. The optimized geometries for 1 ^3^A_1_ of MLCT character and 2 ^3^A_2_ of LMCT character are shown in Figure 5a,b, along with the NTO → NTO transitions associated with the corresponding electronic excitation. The frequency analysis necessary to determine whether the relaxed geometries correspond to local minima or transition states could not be performed due to the lack of analytical second derivatives in the TD-DFT implementation of the ADF software. The optimizations were then performed with the Gaussian16 software, which allows such frequency analysis. The resulting triplet structures obtained with very tight convergence criteria are similar to the ones obtained with ADF and all exhibit imaginary frequencies (S10 in S.I.2). Consequently, the low-lying states of triplet spin multiplicity for each symmetry were also characterized with e-DFT calculations, wherein the occupation of the MOs is constrained and allowed to be non-Aufbau during the self-consistent (SCF) procedure.^46^ The resulting structures of the 2 ^3^A_2_ state with mixed LMCT/MC character and the 3 ^3^A_1_ state with LMCT character are shown in Figure 5c,d. Frequency analyses performed on the optimized geometries indicate that they are actual local minima without imaginary frequencies. The calculations for 1 ^3^A_1_ and 2 ^3^A_1_ converged to extrema corresponding to transition states, with one imaginary frequency. For each state, the MO → MO transition depicts the passing from the closed-shell ground state to the triplet state of interest as a constrained spin-flip promotion (Figure 5c,d). The corresponding spin-density isosurfaces are displayed in S.I.2. Using the D_4h_ notation, which is applicable to planar and quasiplanar geometries, the TD-DFT and e-DFT calculations with C_2v_ constraints identify the 2 ^3^A_2_ triplet state of dominant LMCT (TD-DFT, transition state) or mixed LMCT/MC (e-DFT, minimum) character that implicate an a_2u_-like HOMO and a d_*x*^2^–*y*^2^_-like orbital, thereby noted (a_2u_)^1^(d_*x*^2^–*y*^2^_)^1^. The degenerate 1 ^3^B_1_ and 1 ^3^B_2_ of MC character obtained with TD-DFT and e-DFT are shown in S.I.2 for comparison; although, this character can be ruled out based on the tr-OAS and tr-XES observations.

**Figure 5:**
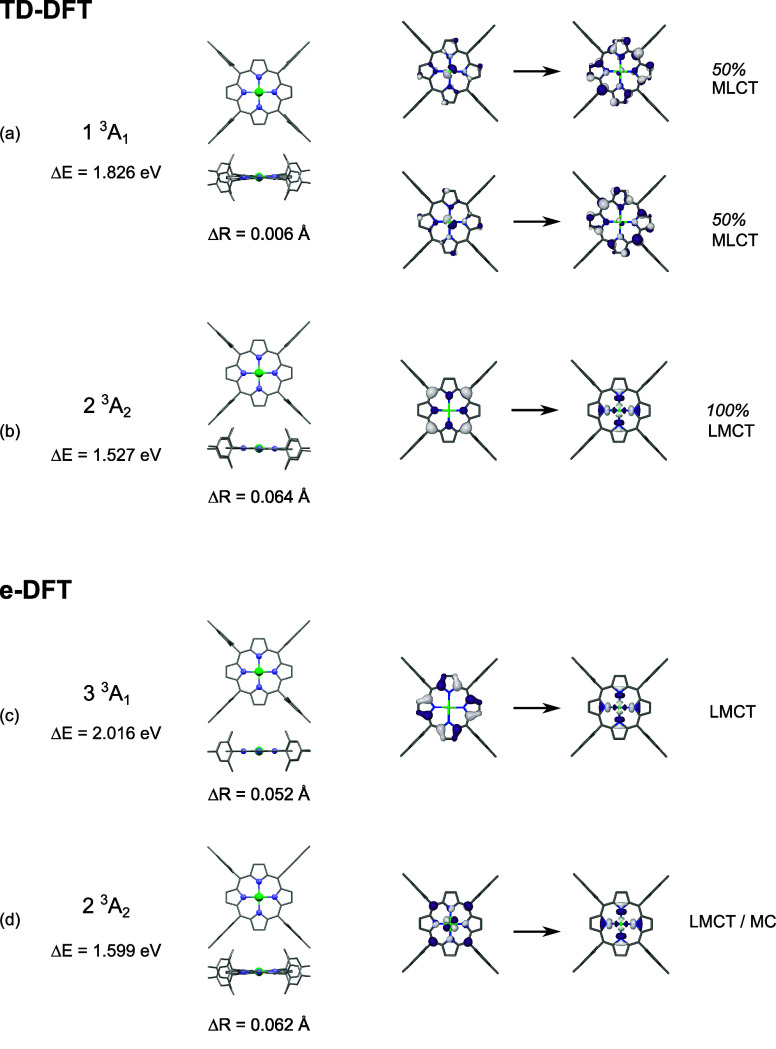
Relaxed TD-DFT structures of the low-lying (a) 1 ^3^A_1_ triplet state of MLCT character and (b) 2 ^3^A_2_ of LMCT character in C_2v_ symmetry. The adiabatic energy difference Δ*E* and the average bond elongation Δ*R* are indicated, along with the main NTO → NTO transitions and their weights. Relaxed e-DFT structures of the low-lying (c) 3 ^3^A_1_ of dominant LMCT character and (d) 2 ^3^A_2_ triplet state of mixed LMCT/MC in C_2v_ symmetry. The adiabatic energy difference Δ*E* and the average bond elongation Δ*R* are indicated, along with the MOs involved in the transitions.

To summarize, following the FC singlet excitation of LC character, i.e., localized on the π-ring, the tr-XES measurements establish that the nonadiabatic formation of the metastable triplet state with MC character, where the excitation is localized over the metal, is mediated by the population of a triplet state with CT character, where the excitation is delocalized over the ligand and the metal. This short-lived state presents a ∼640 fs lifetime. The participation of low-lying CT triplet states in the formation of the MC state is discussed below in connection with the description of the early steps in the photocycle.

By exploiting the synthetic versatility of Ni MPs that enables extensive rational design, systematic investigations using time-resolved electronic and vibrational spectroscopies have delineated generic patterns in their photocycles, which are now summarized. Following photoexcitation to the S_2_ and S_1_ state, the system deactivates very rapidly. Depending upon the experimental conditions (including excitation wavelength, solvent and probing technique), S_2_ and S_1_ are quenched on the subpicosecond time scale, with or without detection of an intermediate species, whose nature (S_1_ or CT), population mechanism (IC or ISC), and lifetime remain elusive.^21−23,29,30,47−50^ On the picosecond time scale, a vibrationally hot (d,d) state, noted (d,d)*, is formed via intramolecular energy relaxation (IVR) and conformational relaxation, as monitored by tr-OAS^47,22,37,38^ and tr-RSS.^33,53,54^ This state thermalizes to an equilibrated (d,d) state through vibrational cooling (VC) over tens of picosecond. The metastable (d,d) state decays back to the ground state on time scales ranging from few hundreds of ps to several ms, via nonradiative channels that are governed by ring-deformation modes, solute–solvent interactions, and temperature.^22,37,38,44^

Combining the findings from the present femtosecond spectroscopic measurements performed in the UV–visible and X-ray spectral ranges with comparable resolution (120 and 110 fs IRF, respectively) yields a comprehensive description of the dynamical processes across the excited-state manifold, along with robust time constants.

Considering first the tr-OAS measurements, the observations follow the general trends outlined above. Photoexcitation into the S_2_ state at 400 nm induces a ^1^(π,π*) transition centered on the π-ring. As seen from Figure 1f and S.I.2, this transition does not contain any appreciable metal-based contribution. In Figure 2a, the absence of any stimulated emission feature similar to the one reported for photoexcited closed-shell porphyrins (containing Mg^2+^ or Zn^2+^) shows that the population of S_1_ is not observed with this technique and that the triplet multiplicity of the excited-state manifold is acquired on the sub-100 fs time scale through ISC. The intricate evolution of the tr-OAS spectral lineshapes (Figure 2a,b) reflects the nonadiabatic intramolecular dynamics induced by the photoexcitation of the π-ring. This stage results in the emergence of a derivative-like feature with a broad absorbance over the red side of the bleach, which evolves to the known spectral profile unambiguously attributed to the equilibrated (d,d) state reached through the VC of the (d,d)* state (Figure 2b,c). The global-fit analysis based on a sequential kinetic model captures this evolution and delivers three time constants of 560 ± 60 fs, 6 ± 1 ps, and 210 ± 20 ps (Figure 2d).

Considering then the tr-XES measurements, photoexcitation into the S_2_ state at 400 nm triggers the IRF-limited population of a triplet state of CT character that presents a well-defined spectral line shape. This state decays to the metastable triplet state of MC character with a time constant of 640 fs. The TD-DFT and e-DFT calculations identify several potential triplet states of CT character (Figure 5). Figure 6 displays the Jablonski diagram of the observable states and the photocycle, where the time constants are established on the basis of the combined (a) optical and (b) X-ray measurements. It highlights the strong complementarity between the two spectroscopies when tracking the formation and the relaxation of the MC state. The energy of the (d,d) state has been estimated in an early measurement based on the transient grating techniques.^55^Figure 6 also suggests similarities and differences between the photocycle of Ni porphyrins and the one of other low-Z transition metal complexes (e.g., square planar Ni complexes and octahedral d^6^ complexes^44^ (see S30 in S.I.8).

**Figure 6:**
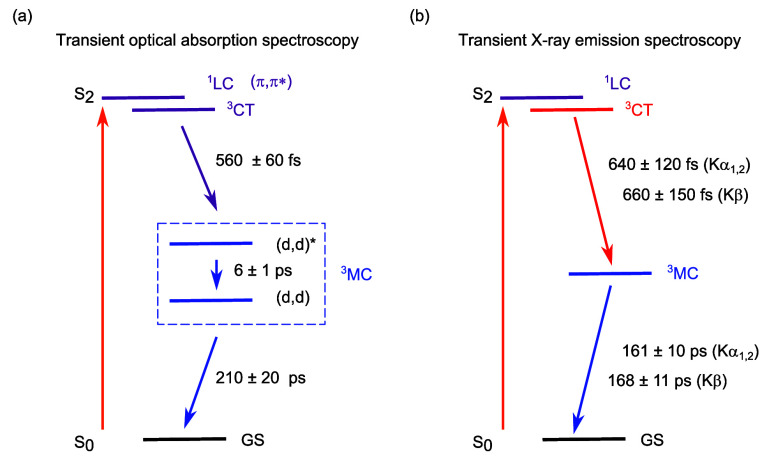
(a) Jablonski diagram and photocycle with (a) the tr-OAS and (b) tr-XES Kα_1,2_ and Kβ time scales.

Additionally, correlating the tr-OAS and tr-XES observations later in the picosecond delay range provides novel insights into the nature of the (d,d)* state. As predicted by the Gouterman model illustrated in Figure 1c, the energetic separation between the HOMO and the HOMO–1 affects the positions and the relative intensities of the B and Q-bands through C.I. and, further, those of the Q(1,0) and Q(0,0) bands through vibronic coupling. Since the spectrum of the equilibrated (d,d) state is very well reproduced by a rigid shift of the GS spectrum without any spectral weight redistribution (Figure 2e), the energetic separation between a_1u_ and a_2u_, the degree of C.I., and the strength of the vibronic coupling are comparable in the GS and in the equilibrated (d,d) state. From Figure 3c,d and 4e,f, the tr-XES measurements show that the (d,d)* state is largely formed by 1 ps. This implies that the corresponding ESA in Figure 2b observed at this particular time delay can already be approximated by the typical UV–vis spectrum comprising B, Q(1,0), and Q(0,0) bands that are red-shifted. As such, the broadened peak detected at 635 nm (red star in Figure 2b) can be identified with the quasi-Q(0,0) band of the (d,d)* state. Based on this tentative assignment, the unexpectedly large energy separation and the relative intensities of Q(1,0) and Q(0,0) evolve during the relaxation from (d,d)* to (d,d) (Figure 2b). This observation then implies that, unlike for the GS and thermalized (d,d) states, the energetic separation between a_2u_ and a_1u_, the degree of C.I., and the strength of vibronic coupling are dynamically altered in the (d,d)* state. In other words, the (d,d)* state should be viewed as a vibronically hot state rather than a simpler vibrationally hot state. At the outset of the photocycle, the dynamics are driven by concurrent ISC, IC, and CT. Elucidating the detailed transition pathway from the singlet FC state to the thermalized triplet (d,d) state will require further experimental studies at higher temporal resolution with assistance from advanced theoretical modeling of the underlying nonadiabatic processes, including vibronic and SOC couplings, while going beyond the sequential kinetic model.

The first key result of this work is that, within a sequential kinetic model, a triplet state of CT character mediates the nonadiabatic intramolecular dynamics, leading to the formation of the metastable triplet (d,d) state of MC character. The CT character of the intermediate triplet species indicates that photoexcited Ni porphyrins can engage in ultrafast intermolecular CT events. Although the lifetime of the CT state is very short, it falls within the characteristic range reported for CT events in MP arrays^56−58^ and MP-containing COFs and MOFs.^59−61^ It is also compatible with the time spans necessary for electron/hole extraction in organic/inorganic nanohybrids of MPs anchored to surfaces (e.g., functionalized electrodes).^62,63^

The second key result of this work is that the thermalization of the (d,d)* state taking place over a few picoseconds involves strong vibronic coupling, which transiently impacts the electronic structure of the molecule, in particular the energetic separation between the a_2u_ and a_1u_ orbitals. This observation suggests that photoexcited Ni porphyrins could conceivably sustain solvent-assisted CT and even vibrationally assisted CT under suitable conditions, provided that energy transfer (EET) to the surroundings can be partly overcome.^64,65^

Some questions still hover over the dynamics of the excited system as it evolves away from the FC region. The sub-100 fs time scale of the CT formation hints at the participation of a conical intersection.^66,67^ However, it is not possible to determine whether ^3^(π,π*) is effectively populated or not. In addition, the strength of the JT effects, which are expected to manifest for all electronically degenerate excited states, cannot yet be assessed.^68^ All these aspects are rooted in the extent of symmetry breaking and the ensuing localization of delocalized electrons and holes, in the initial ground state, intermediate excited states, and metastable state^69^ (Figures 1f, 3g, and 5). Such phenomena could promote the role of other low-lying triplet states, thereby complicating the simplified picture based on quasi-D_4h_ representations.

Ultimately, functionalizing the nonadiabatic intramolecular dynamics of photoexcited Ni porphyrins and other open-shell MPs for boosting the performances of photoconversion in practical applications will rely on maximizing the yield of hot charge separation while minimizing energy losses in optimized architectures. Guidelines for the associated physicochemical engineering will be obtained by establishing structure–property relationships that are applicable on the ultrafast time scale. They will enable correlating the rates of forward/backward hot CT and EET to the nonequilibrated electronic and geometric structures of the short-lived excited species involved in these processes. Owing to the CT character of the manifold mediating the nonadiabatic dynamics, the specific interactions implicating the first solvation shell should be considered for aiding the stabilization of the charge separation.

Elaborating this extended framework will rely on combining ultrafast X-ray emission spectroscopy, X-ray absorption spectroscopy, and diffuse wide-angle and small-angle X-ray scattering in order to provide atomically resolved and spin-resolved kinetics on multiple length scales and time scales. This knowledge will facilitate the interpretation of the multiexponential and probe-wavelength-dependent kinetics that are usually introduced to describe the dynamics of nonadiabatic photoconversion processes in the UV–visible and IR spectral ranges.

Finally, the exploration of the few picoseconds down to the subpicosecond regime is also now within reach for the ultrafast X-ray-based techniques implemented at XFEL facilities and table-top setups. These studies will be assisted by the nonlinear electronic and vibrational laser-based spectroscopies that already reveal ever-finer details about vibronic couplings^70,71^ and by the attosecond methodologies that are currently developed to track charge migration in large organic molecules.^72^

In conclusion, this study probes the early stages of the photoinduced dynamics within the NiTMP molecule in solution using ultrafast tr-OAS and tr-XES achieving comparable femtosecond resolution. Analyzing the two types of measurements by employing a sequential kinetic model with support from TD-DFT and e-DFT calculations reveals that the population of the thermalized metastable state of MC character proceeds via the mediation of a short-lived intermediate state of CT character. This feature should be generally observable in open-shell MPs, setting them apart from closed-shell MPs, where the excited-state manifold remains LC. Besides completing the description of the photocycle for this important representative molecule by delivering robust assignment and time constants, the present findings establish the strong potential of photoexcited Ni porphyrins and other open-shell MPs for joining the classes of low-Z photosensitizers, in which innovative chemical engineering can prolong the lifetime of the CT states. Considering the distinctive formation and deactivation mechanisms outlined in this study, the principles governing the subpicosecond interplay between the CT and LC characters are expected to be specific to this family. Moreover, the vibronically coupled nature of the thermalization dynamics in the MC manifold brings further opportunities for promoting and stabilizing charge separation through the interaction between the building blocks and with the surroundings (solvent or surface) on longer time scales. The comprehensive exploration of the transient properties afforded by the CT manifold of open-shell MPs will require fostering synergies between complementary ultrafast techniques that should be combined in order to realize an exhaustive mapping of the spin, electronic, and structural dynamics on multiple length scales down to the attosecond regime of charge localization and migration. Uncovering the underlying structure-property relationships for the CT manifold of open-shell MPs will lead to the design of nonequilibrated photoreactants that can engage in hot CT steps. Their functionalization will produce novel generations of hybrid molecular architectures capable of boosting the performances reachable by photoconversion schemes out of equilibrium.

## Supporting Information Available

S.I.1. Synthesis of NiTMP, S.I.2. Theoretical calculations and modeling, S.I.3. Transient optical absorption spectroscopy measurements, S.I.4. Transient X-ray emission spectroscopy measurements, S.I.5. Excitation conditions in the transient X-ray emission spectroscopy measurements, S.I.6. Power dependencies, S.I.7. Global spectral analysis of the photoinduced dynamics in the X-ray range, S.I.8. Observation of CT states in low-Z transition metal complexes (PDF)
