# Supplementary material for: Nonadiabatic Charge Transfer within Photoexcited Nickel Porphyrins
Source: J Phys Chem Lett. 2024 Mar 26;15(13):3627–38. doi: 10.1021/acs.jpclett.4c00375 (PMC11000243; doi:10.1021/acs.jpclett.4c00375)
Supplement: Supplementary file 1 — jz4c00375_si_001.pdf [file jz4c00375_si_001.pdf]

Nonadiabatic Charge-Transfer within Photoexcited Nickel Porphyrins

M. A. Naumova, G. Paveliuc, M. Biednov, K. Kubicek, A. Kalinko, J. Meng, M. Liang, A. Rahaman, M. Abdellah, S. Checchia, F. Alves Lima, P. Zalden, W. Gawelda, C. Bressler, H. Geng, W. Lin, Y. Liu, Q. Zhao, Q. Pan, M. Akter, M. Retegan, D. J. Gosztola, K. Kong, M. Pápai, D. Khakhulin, M. Lawson Daku, K. Zheng, and S.E. Canton

Supplementary Information

S.I.1. Synthesis of NiTMP

S.I.2. Theoretical calculations and modeling

S.I.3. Transient optical absorption spectroscopy measurements

S.I.4. Transient X-ray emission spectroscopy measurements

S.I.5. Excitation conditions in the transient X-ray emission spectroscopy measurements

S.I.6. Power dependencies

S.I.7. Global spectral analysis of the photoinduced dynamics in the X-ray range

S.I.8. Observation of CT states in low-Z transition metal complexes

S.I.1. Synthesis of NiTMP

The molecule [5,10,15,20-tetrakis(2,4,6-trimethylphenyl)porphyrinato]nickel(II) (NiTMP) was synthesized as follows. A toluene solution (100 mL) of 5, 10, 15, 20-tetrakis (2, 4, 6- trimethylphenyl)-21h, 23h-porphyrin (400 mg, 0.510 mmol) and Nickel (II) acetate tetrahydrate (317 mg, 1.277 mmol) was heated to reflux for 12 h at 120°C. The completion of the reaction was controlled by thin layer chromatography (TLC). The solvent was removed under reduced pressure and the residue chromatographed by column on silica gel. Elution with CH_2Cl_2: n-hexane (v/v 1:3) developed one band and afforded 5, 10, 15, 20-tetrakis (2, 4, 6- trimethylphenyl)porphyrin Ni(II) as a purple solid in 83 % (356 mg) yield. The characterization data were in accord with those reported earlier. [1, 2]

^{1}H NMR ($CDCl_3$): *δ* 8.53 (s, 8H, *β-CH*), 7.21 (s, 8H, ArH), 2.57 (s, 12H, $-CH_3$) 1.82 (s, 24H, $-CH_3$).

MS: m/z = 841 (MH^+).

References

(1) Synthesis of Highly Twisted and Fully π Conjugated Porphyrinic Oligomers, Satoru Ito, Satoru Hiroto, Sangsu Lee, Minjung Son, Ichiro Hisaki, Takuya Yoshida, Dongho Kim, Nagao Kobayashi, and Hiroshi Shinokubo. *J. Am. Chem. Soc.* **2015**, 137, 142−145.

(2) Ultrafast Excited State Relaxation of a Metalloporphyrin Revealed by Femtosecond X-ray Absorption Spectroscopy, Megan L. Shelby, Patrick J. Lestrange, Nicholas E. Jackson, Kristoffer Haldrup, Michael W. Mara, Andrew B. Stickrath, Diling Zhu, Henrik Lemke, Matthieu Chollet, Brian M. Hoffman, Xiaosong Li, Lin X. Chen. *J. Am. Chem. Soc.* **2016**, 138, 8752−876.

S.I.2. Theoretical calculations and modeling

S.I.2.1 Computational details

The atom labeling used for describing NiTMP is shown in Figure **S1** The H atoms are omitted for clarity.

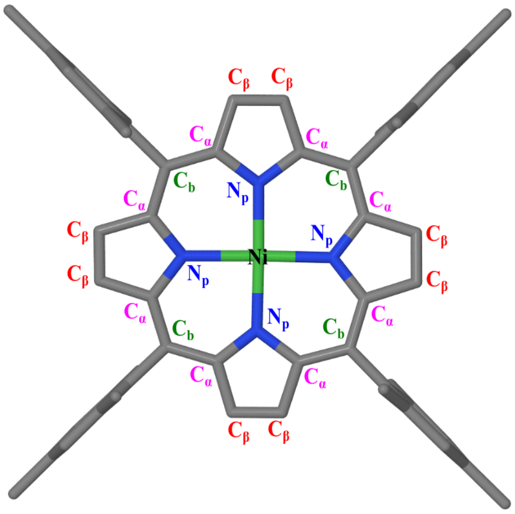

Figure S1 . Atom labeling used for NiTMP (the H atoms are omitted for clarity).

All calculations have been performed with the ADF module of the AMS program package (release 2019.104), [1,2] using the dispersion-corrected PBE-D3BJ functional [3,4] combined with Slater-type (STO) basis sets of triple- ζ polarized quality (TZP) from the ADF basis set database. [5] The atomic core levels were kept frozen up to the 2p level for the Ni atom, and up to the 1s level for the N and C atoms. The calculations were run spin-restricted (resp., spin-unrestricted) for NiTMP in the S_0 closed-shell ground state (resp., triplet open-shell states) and scalar relativistic (SR) effects were included within the zero order regular approximation (ZORA). [6,7] The influence of the toluene solvent were taken into account using the conductor-like screening model of solvation (COSMO). [8,9] The optimizations have been performed with the molecular symmetry constrained to C_{2v}.

Density functional theory (DFT) [10,11] has been applied to the determination of the geometry of NiTMP in toluene in the S_0 ground state and in the metastable T_1 (1^3A_2) state. Frequency analyses performed on the S_0 and T_1 geometries confirmed that the located extrema correspond to true minima (no imaginary frequencies).

For identifying the nature of the initial photoexcitation in the photocycle of NiTMP in toluene, the energies and oscillator strengths of the $S_0 \rightarrow S_n$ (n = 1, 250) electronic transitions have been

computed within linear-response theory in time-dependent DFT (LR-TDDFT),[12,13,14,15] as implemented in ADF [16,17] and using for exchange-correlation kernel the adiabatic local density approximation (ALDA). [18]

Additional LR-TDDFT optimizations have been performed in order to identify local minima on the potential energy surfaces (PESs) of the lowest-lying triplet excited states, which may be populated in the photocycle of NiTMP. The calculations were limited to the search of states lying below the excitation energy (25000 cm^{-1}) and which could thus get populated in the photocycle of the complex. Extrema could thus be found for the 1^3A_2, 1^3B_1, 1^3B_2, and 2^3A_2 states. However, because analytical Hessian evaluation is not implemented, subsequent vibrational frequency analyses could not be performed to ascertain that they correspond to minima.

Extrema on the PESs of the 1^3A_2, 1^3B_1, 1^3B_2, 2^3A_2 and 3^3A_1 triplet excited states could also be characterized within the excited-state DFT (e-DFT) framework [19] : for such calculations, the occupancy of the Kohn-Sham orbital levels has been constrained so as to target triplet states of given spatial symmetries, while allowing for a non-Aufbau occupation of the orbital levels. The vibrational analyses performed on the eDFT-optimized geometries indicate that they are true minima. Extrema could be located for the 1^3A_1 and 2^3A_1 triplets but they correspond to transition states (one imaginary frequency).

The Kohn-Sham orbitals for the singlet ground state are shown in **Figure S2**. The ring-based occupied/unoccupied orbitals are shown in red/orange, while the metal-based occupied/unoccupied orbitals are shown in black/gray.

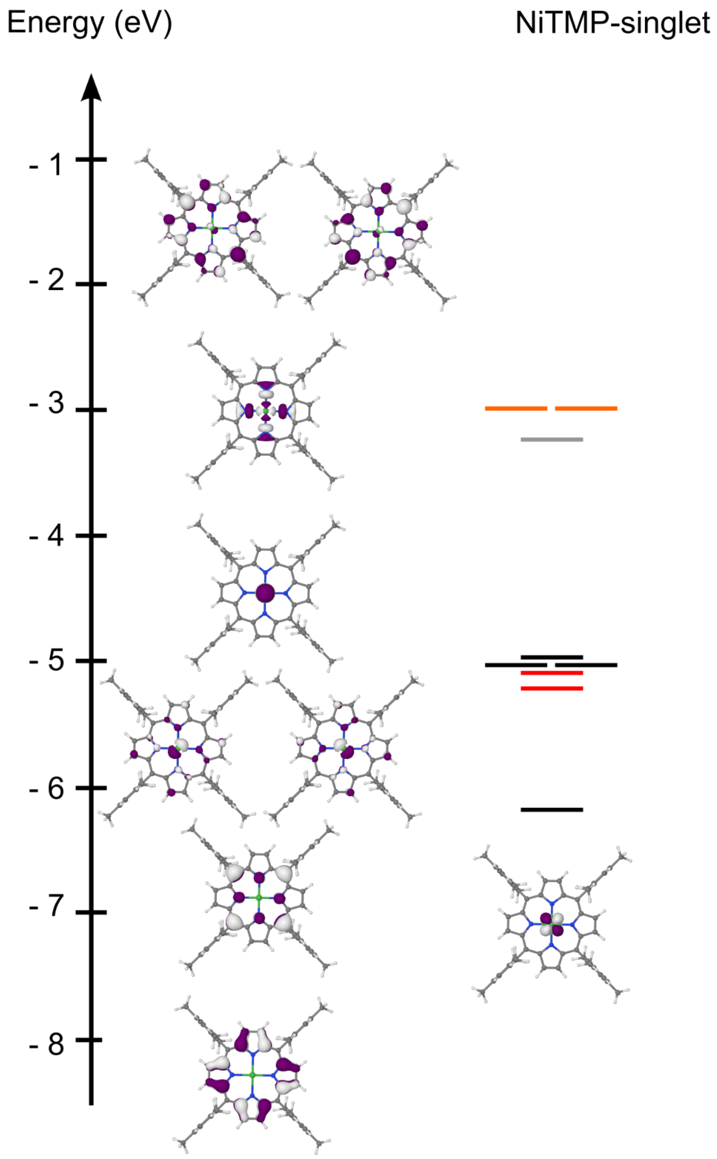

Figure S2. The Kohn Sham orbitals for the singlet ground state of NiTMP in toluene.

S.I.2.2 Predicted ground-state electronic absorption spectrum

Table S3 shows the transition wavelength λ and oscillator strength f of the most intense $S_0 \rightarrow S_n$ transitions ($f \geq 10^{-3}$) in the 350-650 nm range of NiTMP in toluene at its ground-state geometry, along with their analysis in terms of transitions between occupied and virtual natural transition orbitals (NTOs). [20]

Table S3. Wavelength λ and oscillator strength f of the most intense $S_0 \longrightarrow S_n$ transitions ($f \geq 10^{-3}$) in the 350-650 nm range calculated for NiTMP in toluene at its ground-state geometry; the symmetry of the excited state as well as the involved NTO ⟶ NTO transitions and their weights are also reported (LR-TD-SR-PBE-D3BJ/TZP results).

	**Symmetry**	**λ (nm)**	**$10^{-3}\times$f**	**NTO ⟶ NTO transition**			
**S_{10}**	**3^1B_1**	**540**	**4.43**	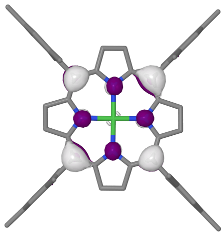	⟶	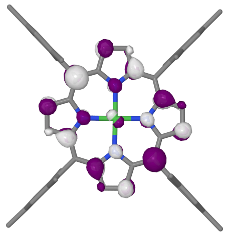	**55.7%**
				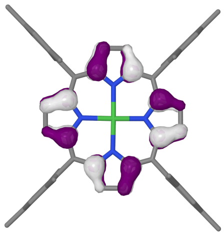		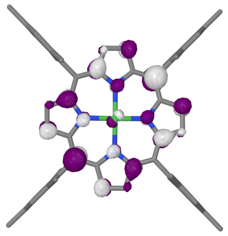	**43.5%**
**S_{11}**	**3^1B_2**	**540**	**4.43**	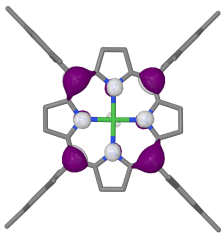		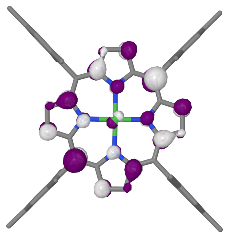	**55.7%**
				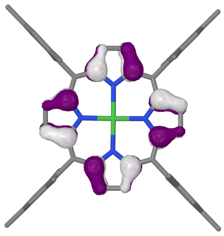		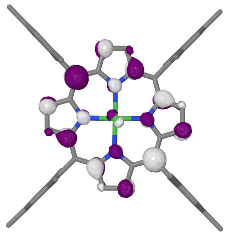	**43.5%**
**S_{15}**	**4^1B_1**	**481**	**1.46**	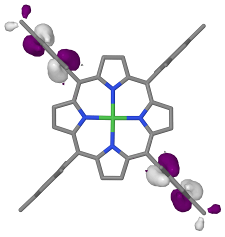		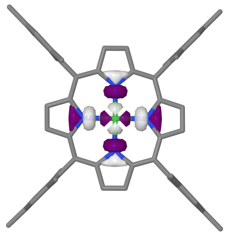	**99.7%**

**S_{16}**	**4^1B_2**	**481**	**1.46**	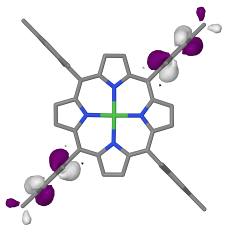		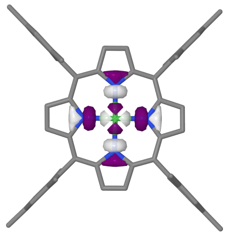	**99.7%**
**S_{28}**	**7^1B_1**	**432**	**14.02**	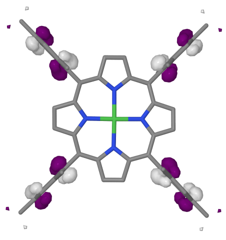		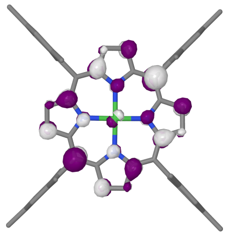	**97.0%**
**S_{29}**	**7^1B_2**	**432**	**14.02**	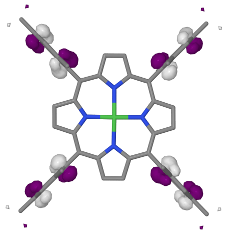		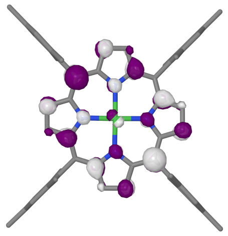	**97.0%**
**S_{30}**	**8^1B_1**	**429**	**910.4**	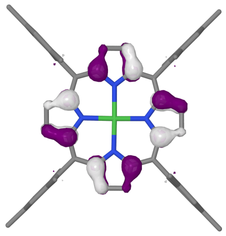		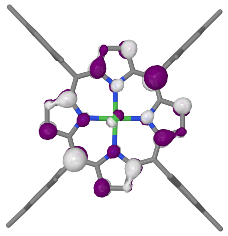	**54.0%**
				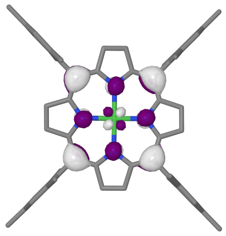		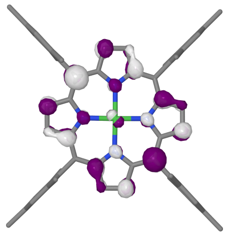	**35.2%**
**S_{31}**	**8^1B_2**	**429**	**910.4**	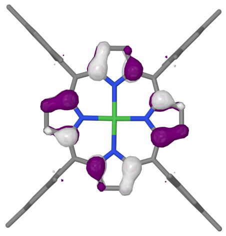		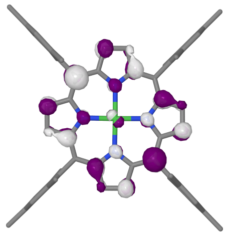	**54.0%**

				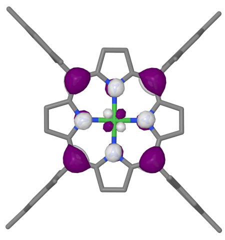		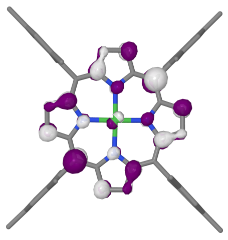	**35.2%**
**S_{34}**	**9^1B_1**	**421**	**8.66**	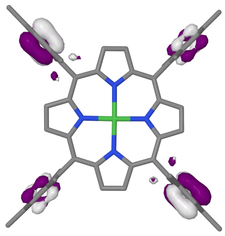		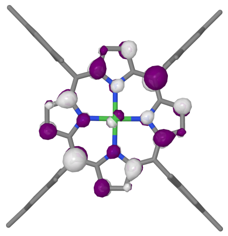	**99.7%**
**S_{35}**	**9^1B_2**	**421**	**8.83**	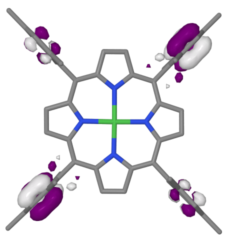		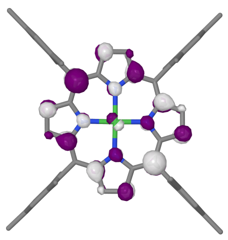	**99.7%**
**S_{38}**	**10^1B_1**	**421**	**37.41**	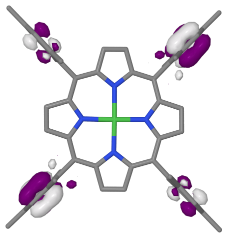		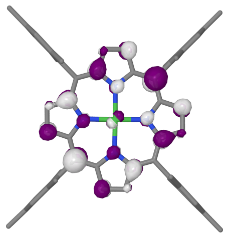	**99.1%**
**S_{39}**	**10^1B_2**	**421**	**37.41**	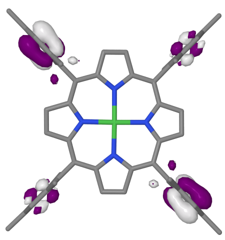		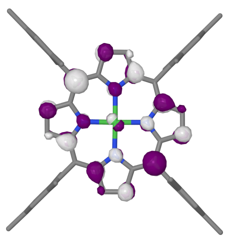	**99.2%**

Figure S4 shows the calculated ground-state electronic absorption spectrum of NiTMP obtained by convoluting the calculated oscillatory strengths with Gaussians having a full width at half maximum (FWHM) of 2000 cm^{-1}. The simulated spectrum satisfactorily reproduces the intense Soret band, which can be ascribed mainly to the $S_0 \rightarrow S_{30}$ and $S_0 \rightarrow S_{31}$ transitions of π-π* character according to the LR-TDDFT results (**Table S3**). However, the low-energy and much weaker Q band could not be reproduced, probably because of the non-inclusion of vibronic coupling in the calculation of the absorption spectrum. [21,22]

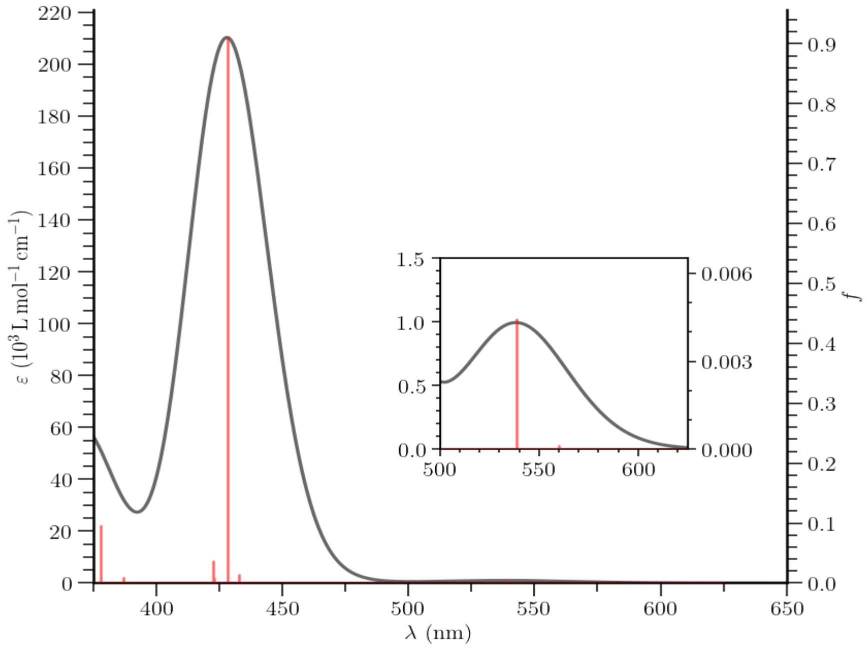

Figure S4. Calculated ground-state UV-Vis absorption spectra of NiTMP in toluene (LR-TD-SR-PBE-D3BJ/TZP results)

S.I.2.3 Optimized triplet geometries of NiTMP in solution

The selected bond lengths and angles characterizing the triplet geometries associated with the extrema identified by LR-TD-DFT optimisations on the 1^3A_2, 1^3B_1, 1^3B_2, 2^3A_2 PESs are summarized in **Table S5**.

Table S5. Selected bond lengths (Angstrom) and angles (deg.) characterizing the local minima identified in the triplet manifold of NiTMP in toluene by LR-TD-DFT optimizations (SR-PBE-D3BJ/TZP results). See **Figure S1** for the atomic labeling scheme.

	1^3A_2	1^3B_1	1^3B_2	2^3A_2
Ni-N_p	2.039	2.021	2.021	2.017
C_α-N_p	1.379	1.381	1.381	1.378
C_α-C_β	1.446	1.443	1.443	1.446
C_β-C_β	1.366	1.367	1.367	1.366
C_α-C_b	1.404	1.401	1.401	1.407
∠ C_α-N_p-C_α	106.5	106.0	106.0	105.0
∠ C_α-C_b-C_α	124.7	124.0	124.0	124.8

The Kohn Sham orbitals for the lowest triplet are shown in **Figure S6.** The ring-based occupied/unoccupied orbitals are shown in red/orange, while the metal-based occupied/unoccupied orbitals are shown in black/gray. The dark red color indicates mixed character of the orbitals.

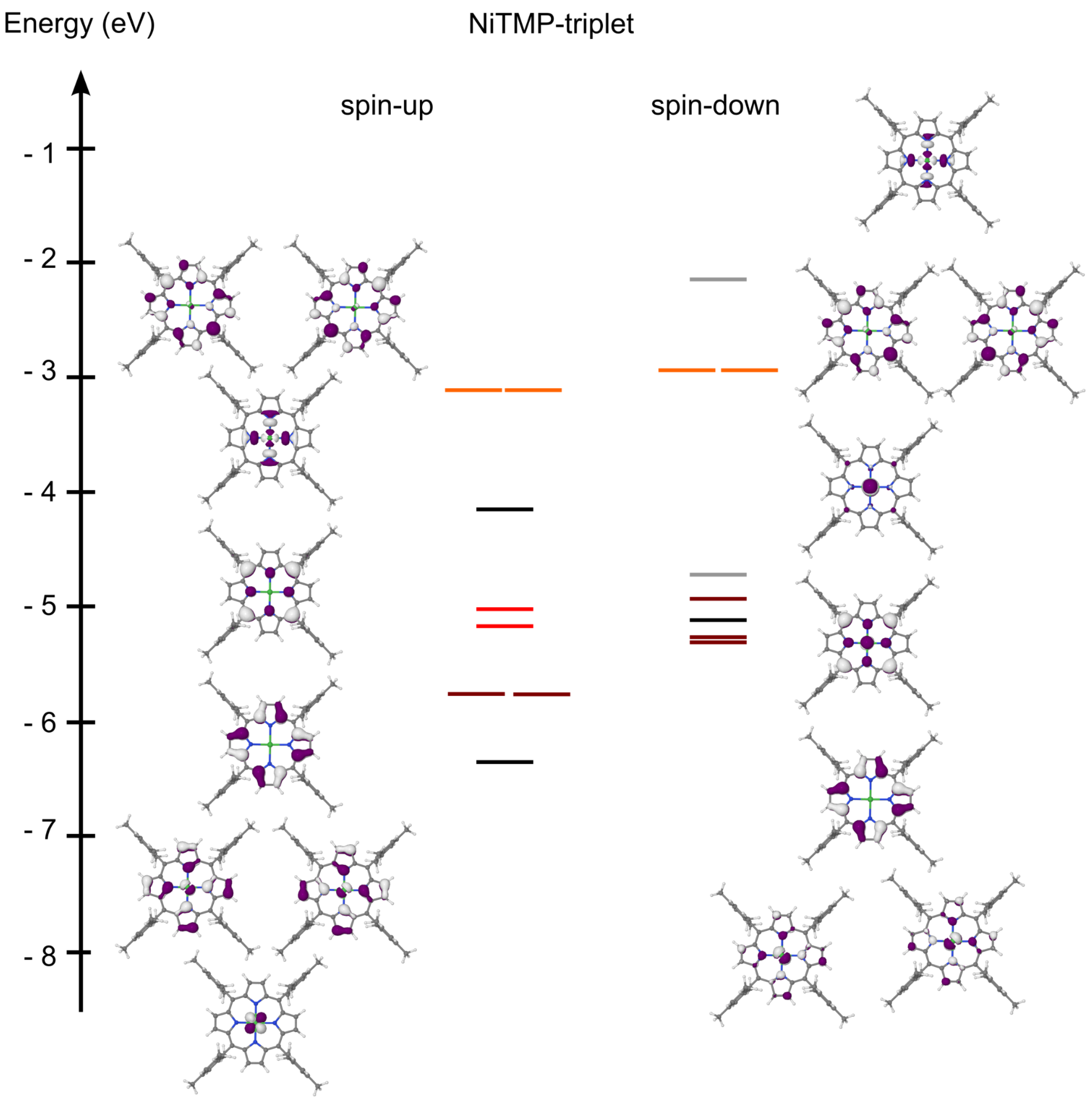

Figure S6. Kohn Sham orbitals for the lowest triplet state of NiTMP in toluene.

Figure S7 summarizes the features of the extrema identified by TD-DFT optimizations in the triplet manifold of NiTMP in toluene. The degenerate 1^3B_1 and 1^3B_2 extrema are of d-d (MC) character like the $T_1 = 1^3A_2$ extremum, while the 2^3A_2 extremum is of LMCT character.

El. state	NTO → NTO	Weight (%)	E_{adia} (eV)	E_{vert} (eV)
1^3A_2	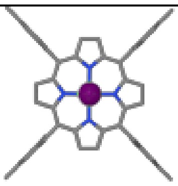 → 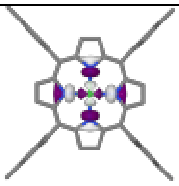	100	0.924	1.242
1^3B_1	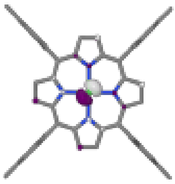 → 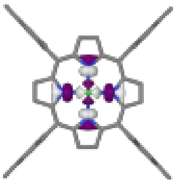	100	1.170	1.394
1^3B_2	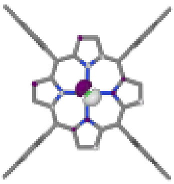 → 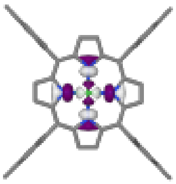	100	1.170	1.394
2^3A_2	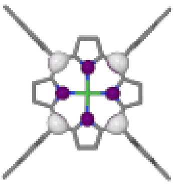 → 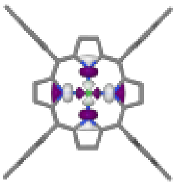	100	1.527	1.937

Figure S7 Features of the extrema identified by LR-TD-DFT optimizations in the triplet manifold of NiTMP in toluene: the NTO → NTO transition associated with transition from the S_0 state to the triplet state at the triple geometry and its weight; the S_0-triplet adiabatic energy difference, E_{adia}, and the S_0-triplet vertical energy difference at the S_0 geometry, E_{vert} (SR-PBE-D3BJ/TZP results).

Table S8 gives selected bond lengths and angles characterizing the triplet geometries associated with the local minima identified by eDFT optimisations on the 1^3A_2, 1^3B_1, 1^3B_2, 2^3A_2 and 3^3A_1 PESs. The 1^3A_2 geometry actually perfectly matches the one of the metastable T_1 d-d state of A_2 symmetry obtained by standard DFT optimization: the two extrema coincide because the constrained occupation of the Kohn-Sham orbital levels in the eDFT optimization is the same as the one obtained by standard DFT optimization.

Table S8 Selected bond lengths (Angstrom) and angles (deg.) characterizing the local minima identified in the triplet manifold of NiTMP in toluene by eDFT optimizations (SR-PBE-D3BJ/TZP results). See **Figure S1** for the atomic labeling scheme.

	1^3A_2	1^3B_1	1^3B_2	2^3A_2	3^3A_1
$Ni\text{-}N_p$	2.026	2.014	2.014	2.015	2.005
$C_\alpha\text{-}N_p$	1.381	1.382	1.382	1.380	1.382
$C_\alpha\text{-}C_\beta$	1.445	1.443	1.443	1.450	1.461
$C_\beta\text{-}C_\beta$	1.366	1.368	1.368	1.366	1.356
$C_\alpha\text{-}C_b$	1.402	1.400	1.400	1.404	1.400
$\angle C_\alpha\text{-}N_p\text{-}C_\alpha$	106.0	105.8	105.8	105.8	105.3
$\angle C_\alpha\text{-}C_b\text{-}C_\alpha$	124.3	123.7	123.7	124.4	123.5

Figure S9 summarizes the features of the local minima identified by e-DFT optimizations in the triplet manifold of NiTMP in toluene. The degenerate 1^3B_1 and 1^3B_2 minima are of d-d (MC) character like the T_1 = 1^3A_2 minimum. The 2^3A_2 minimum exhibits a mixture of d-d and LMCT characters, while the 3^3A_1 minimum is of LMCT character only.

For these triplet states, one can infer from the comparison of the data from **Figure S7** and **S9** that the structures obtained by the LR-TD-DFT and e-DFT optimizations are very similar. Furthermore the nature of these states at the extrema as deduced from the NTO → NTO transitions indicate that the same minima are actually probed by the two approaches.

El. state	MO → MO	Spin density	E_{adia} (eV)	E_{vert} (eV)
1^3A_2	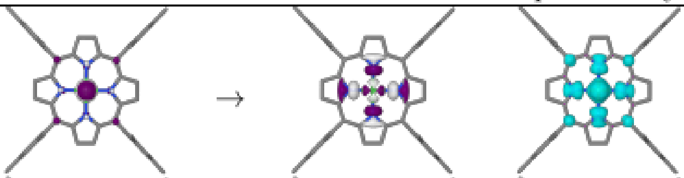		1.163	1.428
1^3B_1	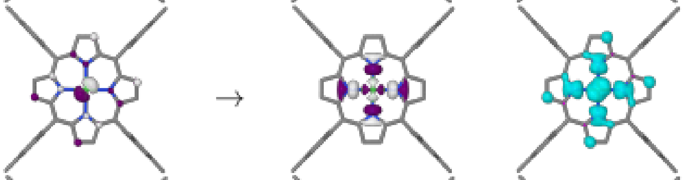		1.166	1.347
1^3B_2	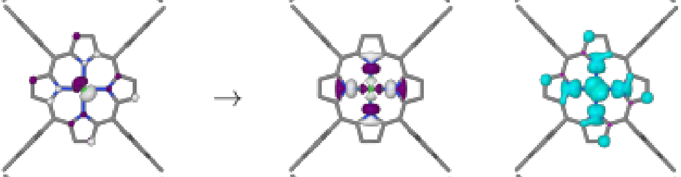		1.166	1.347
2^3A_2	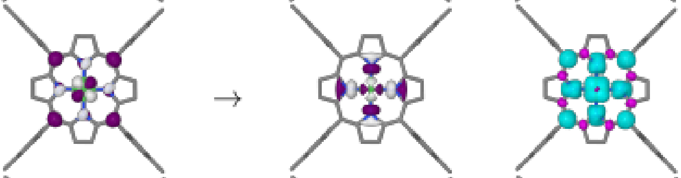		1.599	1.810
3^3A_1	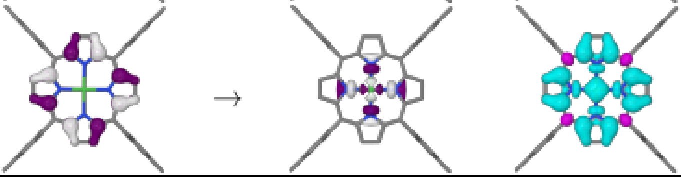		2.016	2.220

Figure S9. Features of the local minima identified by e-DFT optimizations in the triplet manifold of NiTMP in toluene: the MO ⟶ MO transition that helps depict the passing from the closed-shell S_0 state to the triplet state of interest as a constrained spin-flip promotion; the resulting spin density isosurface (0.0008 a.u.); the S_0-triplet adiabatic energy difference, E_{adia}, and the S_0-triplet vertical energy difference at the S_0 geometry, E_{vert} (SR-PBE-D3BJ/TZP results).

References

(1) Velde, G. T.; Bickelhaupt, F. M.; Baerends, E. J.; Guerra, C. F.; Van Gisbergen, S. J. A.; Snijders, J. G.; Ziegler, T. Chemistry with ADF. *Journal of Computational Chemistry* **2001**, *22* (9), 931–967.

(2) ADF 2019.104, SCM, Theoretical Chemistry, Vrije Universiteit, Amsterdam, The Netherlands, http://www.scm.com (last accessed: 2023-06-05). E.J. Baerends, T. Ziegler, A.J. Atkins, J. Autschbach, O. Baseggio, D. Bashford, A. Bérces, F.M. Bickelhaupt, C. Bo, P.M. Boerrigter, C. Cappelli, L. Cavallo, C. Daul, D.P. Chong, D.V. Chulhai, L. Deng, R.M. Dickson, J.M. Dieterich, F. Egidi, D.E. Ellis, M. van Faassen, L. Fan, T.H. Fischer, A. Förster, C. Fonseca Guerra, M. Franchini, A. Ghysels, A. Giammona, S.J.A. van Gisbergen, A. Goez, A.W. Götz, J.A. Groeneveld, O.V. Gritsenko, M. Grüning, S. Gusarov, F.E. Harris, P. van den Hoek, Z. Hu, C.R. Jacob, H. Jacobsen, L. Jensen, L. Joubert, J.W. Kaminski, G. van Kessel, C. König, F. Kootstra, A. Kovalenko, M.V. Krykunov, P. Lafiosca, E. van Lenthe, D.A. McCormack, M. Medves, A. Michalak, M. Mitoraj, S.M. Morton, J. Neugebauer, V.P. Nicu, L. Noodleman, V.P. Osinga, S. Patchkovskii, M. Pavanello, C.A. Peeples, P.H.T. Philipsen, D. Post, C.C. Pye, H. Ramanantoanina, P. Ramos, W. Ravenek, M. Reimann, J.I. Rodríguez, P. Ros, R. Rüger, P.R.T. Schipper, D. Schlüns, H. van Schoot, G. Schreckenbach, J.S. Seldenthuis, M. Seth, J.G. Snijders, M. Solà, M.

Stener, M. Swart, D. Swerhone, V. Tognetti, G. te Velde, P. Vernooijs, L. Versluis, L. Visscher, O. Visser, F. Wang, T.A. Wesolowski, E.M. van Wezenbeek, G. Wiesenekker, S.K. Wolff, T.K. Woo, A.L. Yakovlev)

(3) Perdew, J. P.; Burke, K.; Ernzerhof, M. Generalized gradient approximation made simple. *Physical Review Letters* **1996**, *77* (18), 3865–3868.

(4) Grimme, S.; Ehrlich, S.; Goerigk, L. Effect of the damping function in dispersion corrected density functional theory. *Journal of Computational Chemistry* **2011**, *32* (7), 1456–1465.

(5) Van Lenthe, E.; Baerends, E. J. Optimized Slater-type basis sets for the elements 1–118. *Journal of Computational Chemistry* **2003**, *24* (9), 1142–1156.

(6) Van Lenthe, E.; Baerends, E. J.; Snijders, J. G. Relativistic total energy using regular approximations. *The Journal of Chemical Physics* **1994**, *101* (11), 9783–9792.

(7) Van Lenthe, E.; Ehlers, A. W.; Baerends, E. J. Geometry optimizations in the zero order regular approximation for relativistic effects. *The Journal of Chemical Physics* **1999**, *110* (18), 8943–8953.

(8) Klamt, A.; Schüürmann, G. COSMO: a new approach to dielectric screening in solvents with explicit expressions for the screening energy and its gradient. *Journal of the Chemical Society* **1993**, No. 5, 799–805.

(9) Pye, C. C.; Ziegler, T. An implementation of the conductor-like screening model of solvation within the Amsterdam density functional package. *Theoretical Chemistry Accounts* **1999**, *101* (6), 396–408.

(10) Hohenberg, P. C.; Kohn, W. Inhomogeneous electron gas. *Physical Review* **1964**, *136* (3B), B864–B871.

(11) Kohn, W.; Sham, L. J. Self-Consistent equations including exchange and correlation effects. *Physical Review* **1965**, *140*(4A), A1133–A1138.

(12) *Recent advances in density functional methods*; **1995**.

(13) Stratmann, R.; Scuseria, G. E.; Frisch, M. J. An efficient implementation of time-dependent density-functional theory for the calculation of excitation energies of large molecules. *The Journal of Chemical Physics* **1998**, *109* (19), 8218–8224.

(14) Van Gisbergen, S. J. A.; Groeneveld, J. A.; Rosa, A.; Snijders, J. G.; Baerends, E. J. Excitation energies for transition metal compounds from time-dependent density functional theory. Applications to MnO_4^-, $Ni(CO)_4$, and $Mn_2(CO)_{10}$. *The Journal of Physical Chemistry A* **1999**, *103* (34), 6835–6844.

(15) Casida, M. E.; Huix-Rotllant, M. Progress in Time-Dependent Density-Functional Theory. *Annual Review of Physical Chemistry* **2012**, *63* (1), 287–323.

(16) Van Gisbergen, S. J. A.; Kootstra, F.; Schipper, P. E.; Gritsenko, O. V.; Snijders, J. G.; Baerends, E. J. Density-functional-theory response-property calculations with accurate exchange-correlation potentials. *Physical Review A* **1998**, *57* (4), 2556–2571.

(17) Van Gisbergen, S. J. A.; Snijders, J. G.; Baerends, E. J. Implementation of time-dependent density functional response equations. *Computer Physics Communications* **1999**, *118* (2–3), 119–138.

(18) Bauernschmitt, R.; Ahlrichs, R. Treatment of electronic excitations within the adiabatic approximation of time dependent density functional theory. *Chemical Physics Letters* **1996**, *256* (4–5), 454–464.

(19) Cheng, C.-L.; Wu, Q.; Van Voorhis, T. Rydberg energies using excited state density functional theory. *The Journal of Chemical Physics* **2008**, *129* (12).

(20) Martin, R. L. Natural transition orbitals. *The Journal of Chemical Physics* **2003**, *118* (11), 4775–4777.

(21) Song, Y.; Schubert, A.; Maret, E. L.; Burdick, R. K.; Dunietz, B. D.; Geva, E.; Ogilvie, J. P. Vibronic structure of photosynthetic pigments probed by polarized two-dimensional electronic spectroscopy and ab initio calculations. *Chemical Science* **2019**, *10* (35), 8143–8153.

(22) Reimers, J. R.; Cai, Z.-L.; Kobayashi, R.; Rätsep, M.; Freiberg, A.; Krausz, E. Assignment of the Q-Bands of the chlorophylls: Coherence loss via QX − QY mixing. *Scientific Reports* **2013**, *3* (1).

S.I.2.4 Frequency analysis with Gaussian16 for the 3 lowest triplet states of MLCT, MC and LMCT character

TD-DFT calculations for NiTMP with C_{2v} symmetry constraints using the Gaussian16 software,[1] in which analytical TD-DFT frequencies are implemented For these calculations, we selected three triplet states, one each from the electronic characters ^{3}MC (1 $^3A_2$), ^{3}LMCT (2 $^3A_2$), and ^{3}MLCT (1 $^3A_1$). The geometries of these states were optimized (using very tight tolerance for convergence) starting from the ADF TD-DFT optimized geometries. Analytical frequency calculations were performed for the optimized structures. We used the PBE/TZVP method in conjunction with the D3BJ[2] dispersion correction scheme. Solvation effects were taken into account using the C-PCM model[3] applying toluene as solvent. The Tamm-Dancoff approximation[4] was used for TD-DFT. The calculations were performed at the DTU Computing Center.[5] The main parameters are summarized in **Table S10**.

Table S10. Energy, average Ni-N bond distance noted R and frequencies of the low lying triplet states obtained with Gaussian16.

	**Energy (eV)**	**R (Å)**	**Imaginary Frequencies (cm^{-1})**
1 $^3A_1$ (^{3}MLCT)	1.766	1.944	-574.2597<br>-498.8642<br>-40.4724<br>-40.4676<br>-39.7753<br>-39.7721
1 $^3A_2$ (^{3}MC)	0.821	2.032	-35.2249<br>-35.1917<br>-35.1831<br>-35.1437<br>-4.9036<br>-2.7277
2 $^3A_2$ (^{3}LMCT)	1.510	2.022	-35.2790<br>-35.2084

			-35.1856<br>-35.1511<br>-3.3817<br>-3.3814

References

(1) Gaussian 16, Revision A.03, M. J. Frisch, G. W. Trucks, H. B. Schlegel, G. E. Scuseria, M. A. Robb, J. R. Cheeseman, G. Scalmani, V. Barone, G. A. Petersson, H. Nakatsuji, X. Li, M. Caricato, A. V. Marenich, J. Bloino, B. G. Janesko, R. Gomperts, B. Mennucci, H. P. Hratchian, J. V. Ortiz, A. F. Izmaylov, J. L. Sonnenberg, D. Williams-Young, F. Ding, F. Lipparini, F. Egidi, J. Goings, B. Peng, A. Petrone, T. Henderson, D. Ranasinghe, V. G. Zakrzewski, J. Gao, N. Rega, G. Zheng, W. Liang, M. Hada, M. Ehara, K. Toyota, R. Fukuda, J. Hasegawa, M. Ishida, T. Nakajima, Y. Honda, O. Kitao, H. Nakai, T. Vreven, K. Throssell, J. A. Montgomery, Jr., J. E. Peralta, F. Ogliaro, M. J. Bearpark, J. J. Heyd, E. N. Brothers, K. N. Kudin, V. N. Staroverov, T. A. Keith, R. Kobayashi, J. Normand, K. Raghavachari, A. P. Rendell, J. C. Burant, S. S. Iyengar, J. Tomasi, M. Cossi, J. M. Millam, M. Klene, C. Adamo, R. Cammi, J. W. Ochterski, R. L. Martin, K. Morokuma, O. Farkas, J. B. Foresman, and D. J. Fox, Gaussian, Inc., Wallingford CT, **2016**. MS 2019.104, SCM, Theoretical Chemistry, Vrije Universiteit, Amsterdam, The Netherlands, http://www.scm.com (last accessed: 2023-06-05). R. Rüger, M. Franchini, T. Trnka, A. Yakovlev, E. van Lenthe, P. Philipsen, T. van Vuren, B. Klumpers, T. Soini.

(2) S. Grimme, S. Ehrlich and L. Goerigk, Effect of the damping function in dispersion corrected density functional theory, *J. Comp. Chem*. **2011** *32*, 1456-65.

(3) (a) Barone, V.; Cossi, M. Quantum Calculation of Molecular Energies and Energy Gradients in Solution by a Conductor Solvent Model. *J. Phys. Chem. A* **1998**, *102*, 1995−2001. (b) Cossi, M., Rega, N., Scalmani, G. and Barone, V. (2003), Energies, structures, and electronic properties of molecules in solution with the C-PCM solvation model. J. Comput. Chem. **2003**, 24: 669-681

(4) Hirata, S.; Head-Gordon, M. Time-dependent density functional theory within the Tamm–Dancoff approximation, *Chem. Phys. Lett.* **1999**, *314*, 291-299

(5) DTU Computing Center: DTU Computing Center resources. DOI: 10.48714/DTU.HPC.0001 (**2022**).

S.I.3. Transient optical absorption spectroscopy measurements

Transient optical absorption spectroscopy measurements were carried out on a laser-based spectroscopy setup. A Coherent Legend Ti: Sapphire amplifier (800 nm, 120 fs pulse length, 3 kHz repetition rate) was used. The output is split into pump and probe beams. Excitation pulses at the wavelength of 400 nm were generated using an optical parametric amplifier (Topas C, Light Conversion). The probe pulses (a broad supercontinuum spectrum) were generated from the 800-nm pulses in a CaF_2 crystal and split by a beam splitter into a probe pulse and a reference pulse. The probe pulse and the reference pulse were dispersed in a spectrograph and detected by a diode array. The pump fluence was set to be 1.2×10^{13} photon/cm^2/pulse (6μJ/cm^2/pulse); 1.2×10^{14} photon/cm^2/pulse (60 μJ/cm^2/pulse), and 2.4×10^{14} photon/cm^2/pulse (120 μJ/cm^2/pulse) to guarantee a linear optical regime. The data analysis was performed with the surface Xplorer and the glotaran [1] softwares.

References

(1) Snellenburg, J. J., Laptenok, S., Seger, R., Mullen, K. M., & van Stokkum, I. H. M. Glotaran: A Java-Based Graphical User Interface for the R Package TIMP. *Journal of Statistical Software* **2012**, *49* (3), 1–22.

S.I.4. Transient X-ray emission spectroscopy measurements

Setup description and data acquisition

The transient X-ray emission (TXE) measurements were performed at the FXE instrument of the European XFEL facility [1] in the standard optical pump-X-ray probe configuration for liquid chemistry experiments.[2,3] The setup is shown in **Figure S11**.

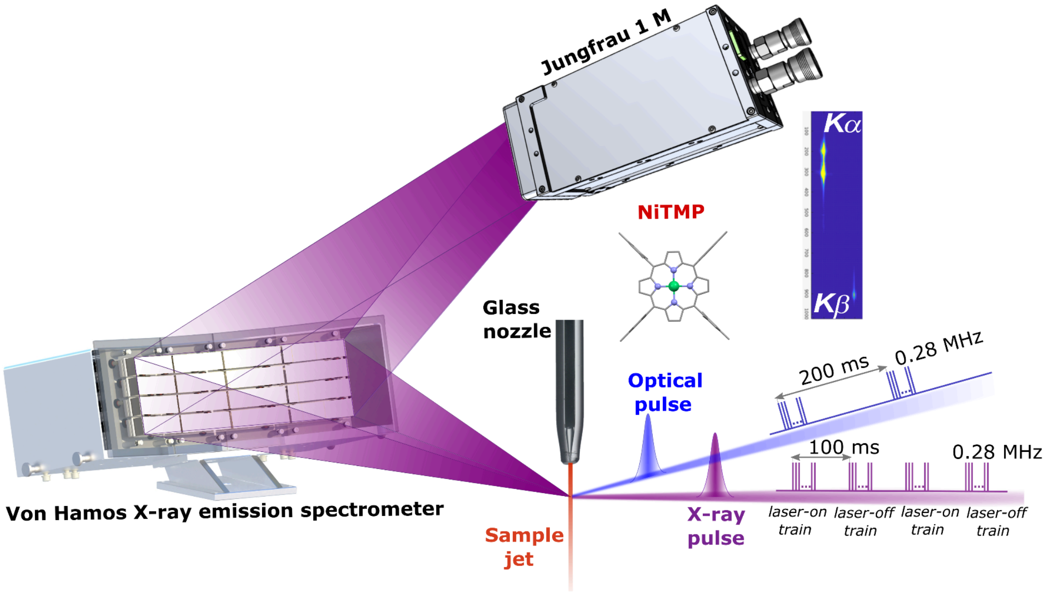

FigureS11. Schematic of the experimental setup for the tr-XES experiment at the FXE instrument of the European XFEL.

The incoming X-ray "pink" beam with SASE spectrum centered at 9.3 keV was focused on the sample jet to a ~10 µm spot (FWHM). The X-ray repetition rate was set to 0.564 MHz with 150 pulses per train. The X-ray pulse energy ~ 2.2 mJ/pulse at the source or ~ 500 µJ/pulse at the sample was used. The Nickel $K\alpha_{1,2}$ and Kβ emission lines were collected by 7 Si(531) and 8 Ge(444) crystals respectively in the von Hamos geometry, which were simultaneously focused and overlapped on a 2D Jungfrau 1M detector running at synchronized 10 Hz frame rate thus acquiring a single accumulated image for each X-ray pulse-train. Optical excitation of sample jets was performed with femtosecond pulses from the standard EuXFEL laser system,[4] which were frequency doubled in a BBO crystal producing 400 nm radiation with pulse duration of ~ 70 fs FWHM. The burst-mode timing structure of laser

pulses was identical to the X-ray pulses except the 5 Hz inter-train repetition rate for the optical laser to ensure the alternation of laser-on and laser-off conditions from train to train. The optical laser beam was focused to a 105 x 87 μm^2 spot (FWHM, horizontal x vertical) as measured at the sample position.

The pump and the probe beam were overlapped in time and space on a flowing liquid round jet of 100 µm diameter. For such conditions the experimentally determined width of temporal instrument response function was ca. 110 fs FWHM.[2] The jet linear speed was adjusted to ca. 60 m/s to ensure a refreshed spot for each pump-probe event, which was verified by measuring the correlation of the incoming intensity to the XES signal strength for different number of pulses as described elsewhere.[5] The concentration of NiTMP was ~ 4 mM in toluene. No sample damage was observed either in the laser-off XES spectra, or in the UV-vis absorption spectra taken before/after the X-ray measurements.

References

(1) A. Galler, W. Gawelda, M. Biednov, C. Bomer, A. Britz, S. Brockhauser, T.-K. Choi, M. Diez, P. Frankenberger, M. French, D. Görries, M. Hart, S. Hauf, D. Khakhulin, M. Knoll, T. Korsch, K. Kubicek, M. Kuster, P. Lang, F. Al. Lima, F. Otte, S. Schulz, P. Zalden, C. Bressler, *J. Synchrotron Radiat.* **2019**, *26*, 1432–1447.
(2) D. Khakhulin, F. Otte, M. Biednov, C. Bömer, T.-K. Choi, M. Diez, A. Galler, Y. Jiang, K. Kubicek, F. A. Lima, A. Rodriguez-Fernandez, P. Zalden, W. Gawelda, C. Bressler, *Appl. Sci.* **2020**, *10*, 995.
(3) F. A. Lima, F. Otte, M. Vakili, F. Ardana-Lamas, M. Biednov, F. Dall'Antonia, P. Frankenberger, W. Gawelda, L. Gelisio, H. Han, X. Huang, Y. Jiang, M. Kloos,, T. Kluyver, M. Knoll, K. Kubicek, I. J. Bermudez Macias, J. Schulz, O. Turkot, Y. Uemura, J. Valerio, H. Wang, H. Yousef, P. Zalden, D. Khakhulin, C. Bressler and C. Milne *J. Synchrotron Rad.* **2023**, *30*, 1168-1182.
(4) G. Palmer, M. Kellert, J. Wang, M. Emons, U. Wegner, D. Kane, F. Pallas, T. Jezynski, S. Venkatesan, D. Rompotis, E. Brambrink, B. Monoszlai, M. Jiang, J. Meier, K. Kruse, M. Pergament and M. J. Lederer, *J. Synchrotron Rad.* **2019**, *26*, 328-332.
(5) M. Biednov, H. Yousef, F. Otte, T.-K. Choi, Y. Jiang, P. Frankenberger, M. Knoll, P. Zalden, M. Ramilli, W. Gawelda, S.E. Canton, F. Alves Lima, C. Milne, D. Khakhulin, *Nucl. Inst. Methods Phys. Res. A* **2023** *1055*, 168540.

Data extraction

The XES spectra for laser-on and laser-off trains were extracted from individual images by selecting a suitable region of interest (ROI) around the spectral lines, $K\alpha_{1,2}$ or Kβ. The width of such ROIs was 6 ($K\alpha_{1,2}$) and 10 (Kβ) detector pixels in the focusing direction of analyzer crystals, since all emission spectra from a respective set of crystals were overlapped in one region. In order to reduce potential influence of a varying elastic scattering background on the spectral lineshapes, two background regions with identical size were set on two sides of the emission spectra for each of the lines, then averaged and subtracted from the spectra themselves. The resulting individual spectral ROIs were

averaged in the non-dispersive direction resulting in spectral curves for each train. The spectra with weakest signals were filtered out and then sorted into the laser-on and laser-off sets and according to the values of varied experimental parameters, e.g. pump-probe delay or optical excitation fluence setting. The sorted spectra were normalized to the total integrated intensity. All laser-off spectra were summed. All the data were checked for the potential detection non-linearity that was corrected when necessary according to the published procedure.[1]

We observed that positions of peaks on the detector changed slightly in the course of the beamtime as a result of minor changes in the source point due to occasional small drifts of the direct beam position on the jet. The shift was below one pixel. One pixel corresponds to ~0.21 eV. In order to correct for this, the center of mass of Kα_1 (as the strongest peak) was calculated for laser-off spectra for each measurement (run). One good-quality laser-off spectrum was chosen as a reference and all other spectra from the beamtime were shifted by the difference in position of their center of mass relative to the reference center of mass, and rebinned to the same grid (pixels in this case). The same shift in pixel units was applied to K$\alpha_{1,2}$ and Kβ spectra.

The kinetic dataset with 0.81 µJ/pulse optical pump required special treatment. In this case, peak shift started to occur in the course of the scan, likely due to a vertical drift in the X-ray beam position. In order to correct it, laser off spectrum for each time delay in the scan was separately shift-corrected (the corresponding laser-on spectra were corrected by the same shift). Then all laser off spectra were summed. Further processing was the same as for other runs.

References

(1) M. Biednov, H. Yousef, F. Otte, T.-K. Choi, Y. Jiang, P. Frankenberger, M. Knoll, P. Zalden, M. Ramilli, W. Gawelda, S.E. Canton, F. Alves Lima, C. Milne, D. Khakhulin, *Nucl. Inst. Methods Phys. Res. A* **2023** *1055*, 168540.

Energy calibration of X-ray emission spectra

Energy calibration of FXE spectra was performed using externally calibrated spectrum of NiTMP acquired at the P64 beamline of Petra III (1). The same calibration was performed to all data sets.

First, linear background was subtracted from the extracted spectra *vs* pixels in the energy region of K$\alpha_{1,2}$ lines (7444-7494 eV) or Kβ line (8229-8290 eV) for corresponding datasets.

For $K\alpha_{1,2}$, after correspondence of pixel number and energy was established for both $K\alpha_1$ and $K\alpha_2$ peaks for NiTMP, other pixels were calibrated to their energy values following planimetric rules for a von Hamos geometry spectrometer.

For Kβ, correspondence of pixel number and energy was established for the maximum of the Kβ line for NiTMP. Further calibration of pixels to energy was done following planimetric considerations for a von Hamos geometry spectrometer and assuming that the height drawn from the center of the crystal array to the line connecting the sample and the detector is 500 mm as specified by crystal radii.

The background-subtracted and energy-calibrated spectra were further individually normalized by their total areas.

The transient signal was calculated as the difference between laser-on and laser-off spectra obtained as described above.

Correction of timing drifts

The possibility of time drifts was considered. The value of t_0 was determined by fitting fine kinetic scans and no further correction was applied.

References

(1) A. Kalinko, W. A. Caliebe, R. Schoch and M. Bauer, *J. Synchrotron Rad.* **2020**, *27*, 31-36.

S.I.5 Excitation conditions in the transient X-ray emission spectroscopy measurements

The tr-XES measurements were conducted under a set of different pump laser powers summarized in **Table S12**. Both lineshapes and kinetics (long-range and short-range) were acquired.

Table S12. Excitation conditions for tr-XES study. All numbers are given per one pump laser pulse.

Measurement	Pulse energy [μJ]	Fluence [mJ/cm2]	Photons per molecule	Photons per cross-section
low power	0.36	1.8	0.5	2.0
high power	0.81	3.9	1.1	4.4

The laser power was measured using a power meter placed before the focusing lens (last optical element before the sample) during the alignment. Transmission through the focusing lens was ~ 70% at 400 nm, so the power at the sample was scaled by 0.7 relative to the measured value. The laser power was controlled by rotating a polarizer. The output power was measured for different positions of the polarizer angle and fitted with a $\cos^4$ function in order to determine the laser power for all polarizer positions. The pulse energy was calculated as the laser power divided by the laser repetition rate. The laser beam was approximated as a cylinder with a diameter equal to the mean of two ellipse axes. The laser-illuminated area for average flux was calculated as $S = \pi w_1 w_2$, while for the peak flux (intensity on the beam axis) as $S = \pi w_1 w_2/2$, where w_1 and w_2 are beam radii at $1/e^2$ of the maximum intensity. The laser-illuminated volume was calculated using the formula for intersecting cylinders. Since the laser beam cross-section was an ellipse and not a circle, the laser beam was approximated as a cylinder with a diameter equal to the mean of two ellipse axes. The number of molecules in the illuminated volume was calculated based on optical density measured either in advance with a laboratory UV-vis spectrometer or in parallel with TR-XES using a flow-through cuvette and a modular UV-vis spectrometer. The fluence and the number of photons per molecule are given for average flux. The number of photons per cross-section is given for peak flux. The number of photons per cross-section does not depend on the sample concentration. Given the many assumptions introduced in the calculations, the numbers in **Table S12** can only serve to approximately give an idea of the excitation conditions.

References

(1) Common volume of two intersecting cylinders. *Journal of research of the National Bureau of Standards -- C. Engineering and Instrumentation* **1965** 69C-2, 139-143.

S.I.6 Power dependencies

In order to study response of the system under different pump laser powers, power dependencies were acquired at different time delays. Similarly to 1D kinetic traces, the absolute values of the sum of counts under each part of Ka_1 transient were summed to obtain a figure of merit (FOM). These 1D traces are shown on **Figures S13** and **S14**. These figures show that, at first, the transient area grows approximately linearly and then it reaches saturation.

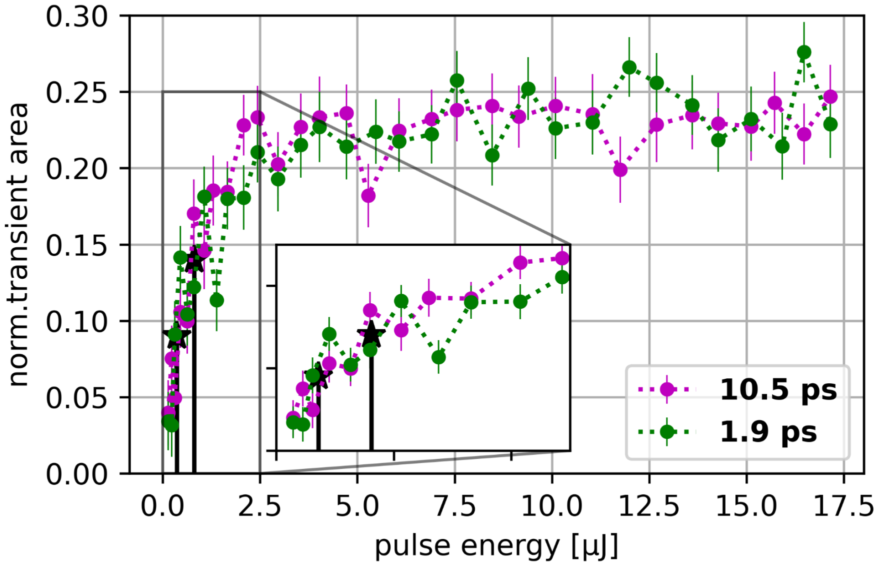

Figure S13. Power dependency. The two black stars mark 0.36 and 0.81 μJ, where two kinetic datasets were taken as reported in **S.I.5**. The inset shows the 0-2.5 μJ window.

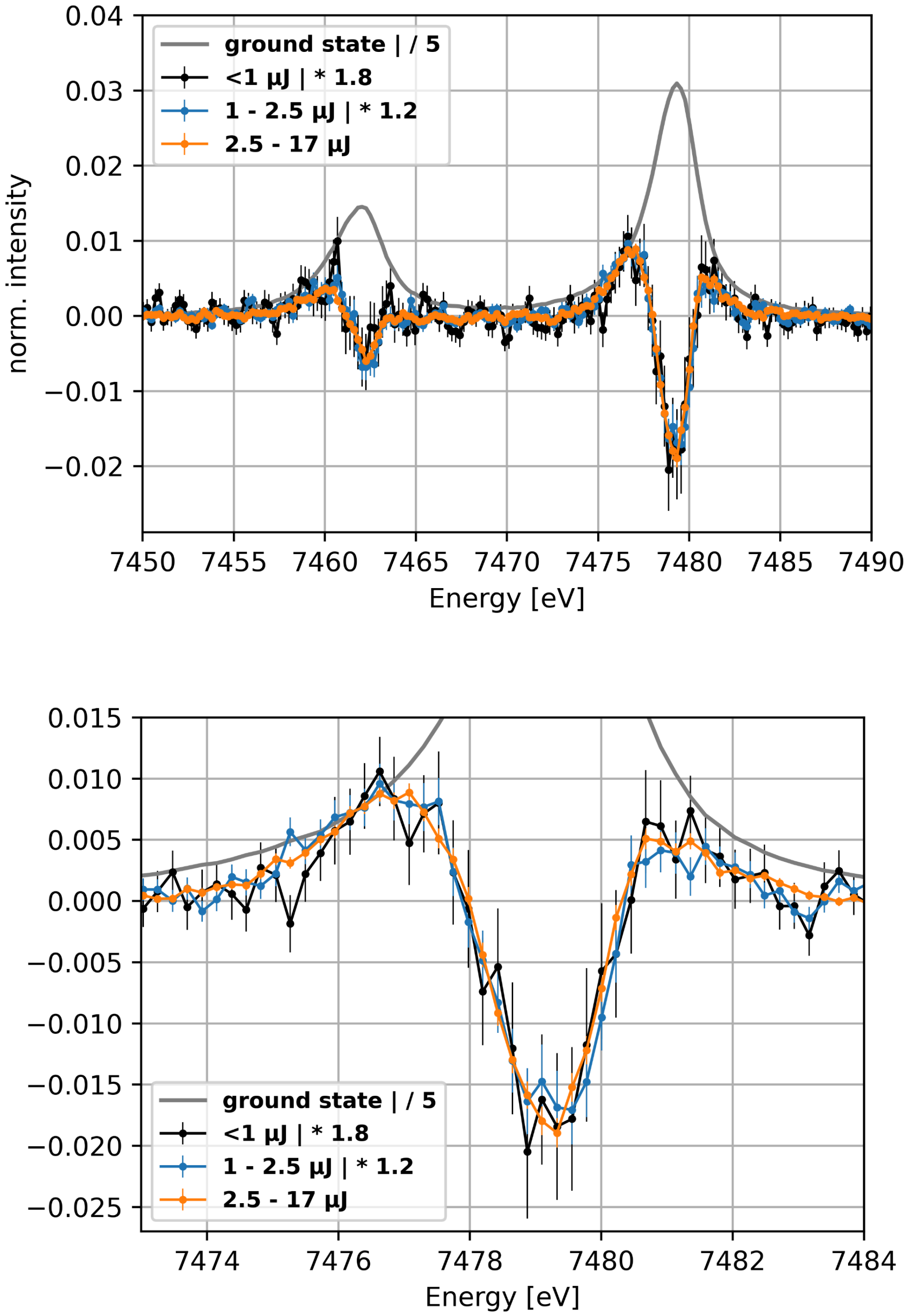

Figure S14. Mean of transient spectra measured at different pump laser pulse energies for a time delay between laser and X-ray pulses of 10.5 ps. The spectra are scaled to match their amplitudes for better visual comparison. Top: K$\alpha_{1,2}$ spectra; Bottom: zoom into Kα_1 region. The data correspond to the magenta trace on **Figure S13.**

S.I.7 Global spectral analysis of the photoinduced dynamics in the X-ray range

Examining the lineshapes of the tr-XES spectra at different time delays between laser and X-ray shows that there are at least two different components in the spectra. Thus, a biexponential sequential decay model convoluted with Gaussian IRF function was used for fitting the spectral evolution as a function of time.

The formulas for spectral contributions of the first and second components C1 and C2 are then:

C1:

$$\frac{1}{2} \cdot a_1 \cdot erfc\left(\frac{\sigma_{pr}}{\sqrt{2}\tau_1} - \frac{t-t_0}{\sqrt{2}\sigma_{pr}}\right) \cdot exp\left(-\frac{t-t_0}{\tau_1}\right)$$

C2:

$$\frac{1}{2} \cdot a_2 \cdot erfc\left(\frac{\sigma_{pr}}{\sqrt{2}\tau_2} - \frac{t-t_0}{\sqrt{2}\sigma_{pr}}\right) \cdot exp\left(-\frac{t-t_0}{\tau_2}\right) - \frac{1}{2} \cdot a_2 \cdot erfc\left(\frac{\sigma_{pr}}{\sqrt{2}\tau_1} - \frac{t-t_0}{\sqrt{2}\sigma_{pr}}\right) \cdot exp\left(-\frac{t-t_0}{\tau_1}\right)$$

where a_1, τ_1, a_2, τ_2 are the amplitudes and the lifetimes of C1 and C2, respectively; σ_{pr} is the residual mean square (rms) of the Gaussian IRF; t_0 is time-zero.

In order to determine the lifetimes and the spectral lineshapes of the excited species, both 1D kinetic fits (figure of merit (FOM) vs time) and 2D (energy, time) global fits were performed. For 1D fits, the summed absolute value of the integrated areas under the transient difference signals was used as FOM. Comparing their results shows that both agree with the same model of a two-component sequential exponential fit (where one exponent was fixed to 200 ps).

The data fits were done using lmfit python package:

https://lmfit.github.io/lmfit-py/

The global fit variables are presented in **Table S15.**

Multiexponential fits can have strong correlations between fit parameter values; therefore it was important to do further statistical analysis of fit results. Confidence intervals for τ_1 were estimated using an F-test:

https://lmfit.github.io/lmfit-py/confidence.html

Table S15. Fit variables in global fits.

global		non-global
**fixed**	**fitted**	
τ_2 = 200 ps (from this study)<br>Rms of the IRF: σ_{pr} = 47 fs (FWHM = 110 fs; fixed based on report by FXE and own fits of fine rise scans).	τ_1<br>t_0	a_1, a_2: for each energy point (pixel on the detector) value of amplitude was fitted. It formed spectral lineshape of first and second components C1 and C2.

S.I.7.1. Long-range kinetics

Long-range kinetic scans were done at two excitation powers, 0.36 and 0.81 µJ (see **Table S12**). These scans allowed to determine the lifetime of the metastable state of NiTMP.

Fit results for these traces are given in **Table S16** and **Table S17** and data and fits are plotted in **Figure S18.**

A single-exponential decay convolved with a Gaussian IRF was used as a fitting model:

$$\frac{1}{2} \cdot a \cdot erfc\left(\frac{\sigma_{pr}}{\sqrt{2}\tau} - \frac{t-t_0}{\sqrt{2}\sigma_{pr}}\right) \cdot exp\left(-\frac{t-t_0}{\tau}\right) + y_0$$

where a, τ are amplitude and lifetime of the metastable state; σ_{pr} is the residual mean square (rms) of the Gaussian IRF; t_0 is time-zero; y_0 is an offset.

Three variations of this model were tested:

1) fitting of decay+rise, with offset fixed to the mean of the values before t_0

2) fitting of decay (without rise), with offset fixed to the mean of the values before t_0

3) fitting of decay+rise, with offset fitted

For models where the rise region was included, t_0 was fixed to the best-fit value obtained in a fine time delay scan performed shortly before these two scans. IRF FWHM was fixed to 110 fs. Fits with different models result in almost identical curves and similar values for τ.

Table S16. Lifetime of the metastable state τ [ps] obtained from fits for "low power" long kinetic traces (**0.36 µJ**).

	**Fitting model**		
	**Fit 1** Rise + decay is fitted, offset fixed to mean of values before the rise	**Fit 2** Only decay is fitted	**Fit 3** Rise + decay is fitted, offset is fitted
$K\alpha_1$	181 ± 19	181 ± 22	195 ± 29
$K\alpha_2$	112 ± 21	112 ± 22	135 ± 31
$K\alpha_{1,2}$	161 ± 16	161 ± 18	177 ± 25
Kβ	143 ± 31	143 ± 34	142 ± 45

Table S17. Lifetime of the metastable state τ [ps] obtained from fits for "high power" long kinetic traces (**0.81 µJ**).

	**Fitting model**		
	**Fit 1** Rise + decay is fitted, offset fixed to mean of values before the rise	**Fit 2** Only decay is fitted	**Fit 3** Rise + decay is fitted, offset is fitted
$K\alpha_1$	183 ± 12	183 ± 14	191 ± 19
$K\alpha_2$	115 ± 12	115 ± 13	127± 18
$K\alpha_{1,2}$	161 ± 10	161 ± 11	169 ± 16
Kβ	168 ± 11	168 ± 11	173 ± 17

The value of τ_0 was determined from the global fit of short-range dataset with 0.36 µJ excitation, as described in section **S.I.7.2**. The best-fit value for $K\alpha_1$ fit was t_0 = 3591.514 +/- 0.017 ps (time scale is determined by the phase shifter and the shift on the scale is arbitrary), and this value was subtracted from time axis of the kinetic scans.

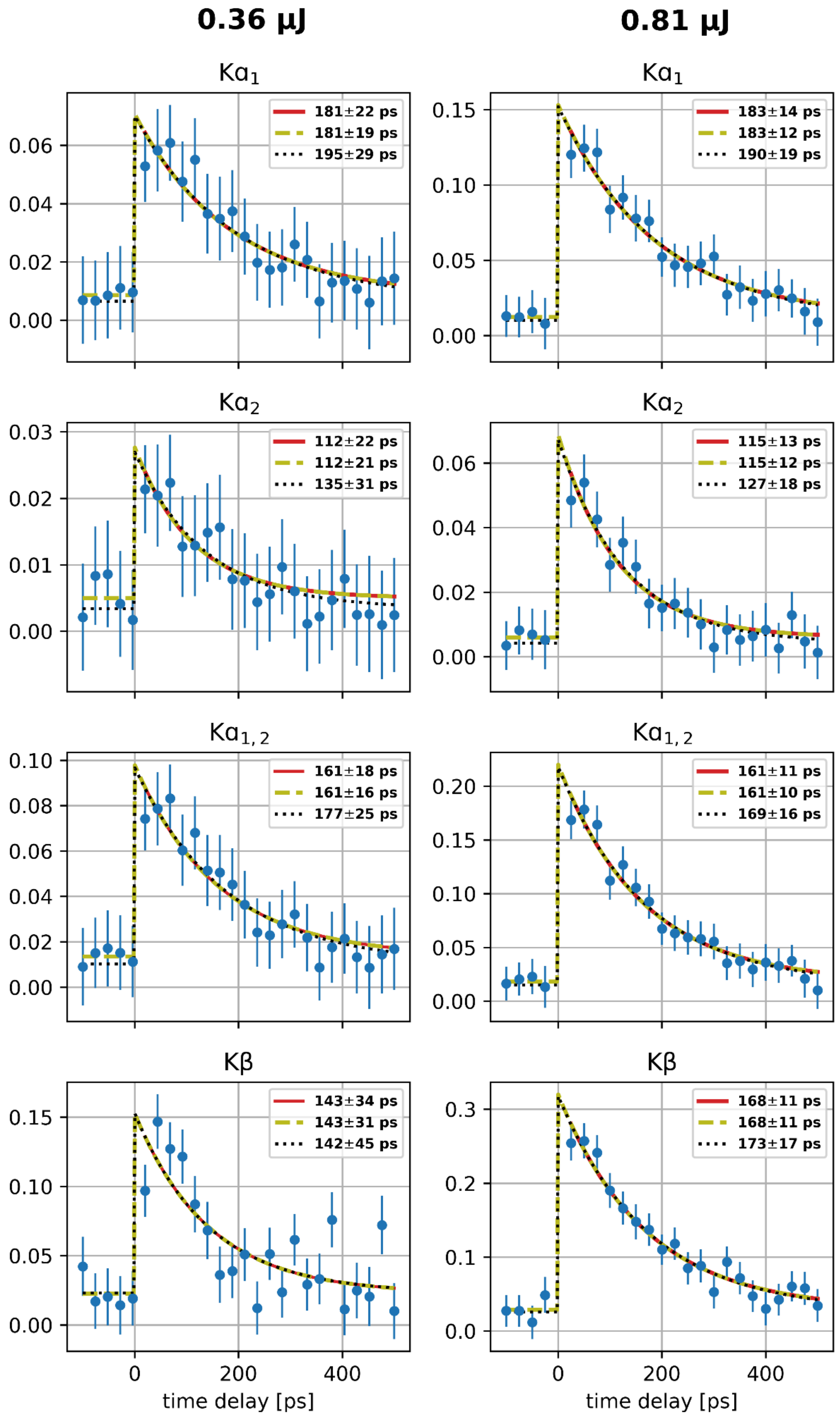

Figure S18. Long kinetics for laser pump pulse energies of **0.36** and **0.81 μJ**. Fit models are described in the text.

S.I.7.2. Short-range kinetics

Transient spectra as a function of time delay in a short-range (~ -1 to 2 ps) were measured with optical laser pulse energy of **0.36 μJ** and **0.81 μJ**.

In order to determine the lifetime of the intermediate state, the data were fitted as described in the beginning of section **S.I.7**. During the fits τ_2 was fixed to 200 ps, delivered by the kinetics measurement over the long range of time delays. Tests showed that fixing τ_2 in 150-250 ps range did not affect the value of τ_1 obtained by fitting data in -1 to 2 ps window.

The lineshapes of the components C1 and C2 obtained in the global fits are shown on **Figure S19** (separately Kα_1 and Kα_2) and Figure **S20** (K$\alpha_{1,2}$ and Kβ).

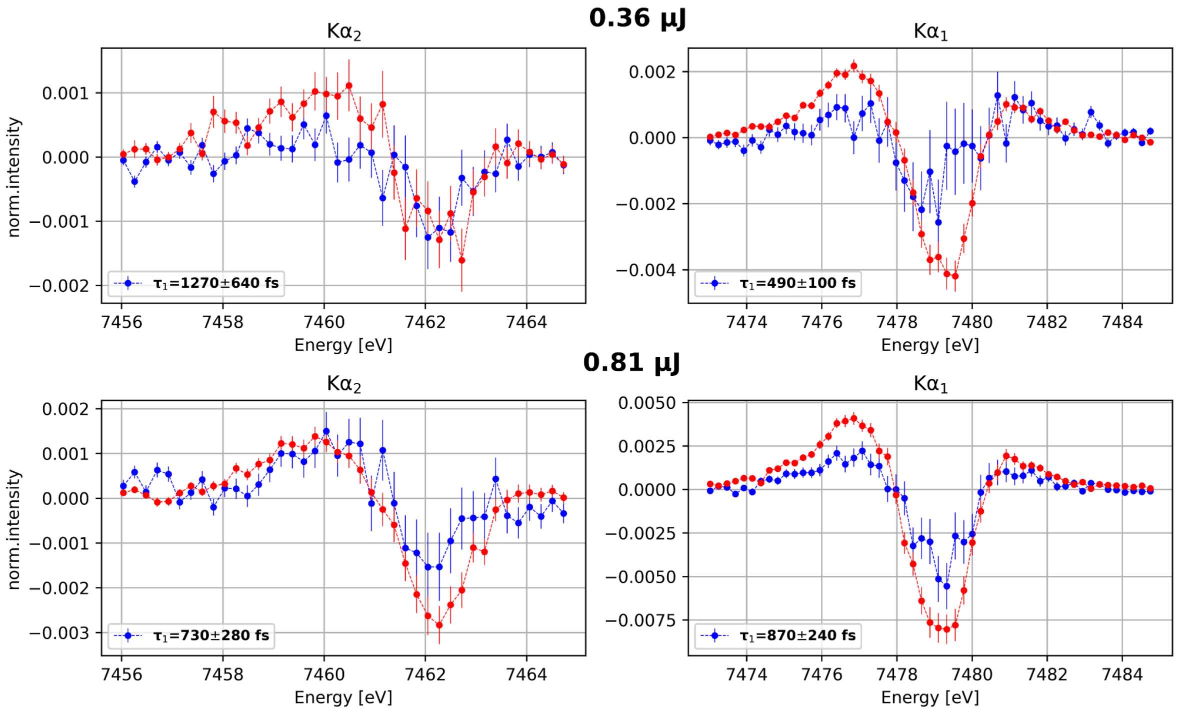

Figure S19. Global fit results for excitation with 0.36 and 0.81 μJ pump laser pulses. Left column: Kα_2; right column: Kα_1. Kα_1 and Kα_2 were fitted separately. t_0 for Kα_2 was fixed to the best-fit value obtained in global fit of Kα_1.

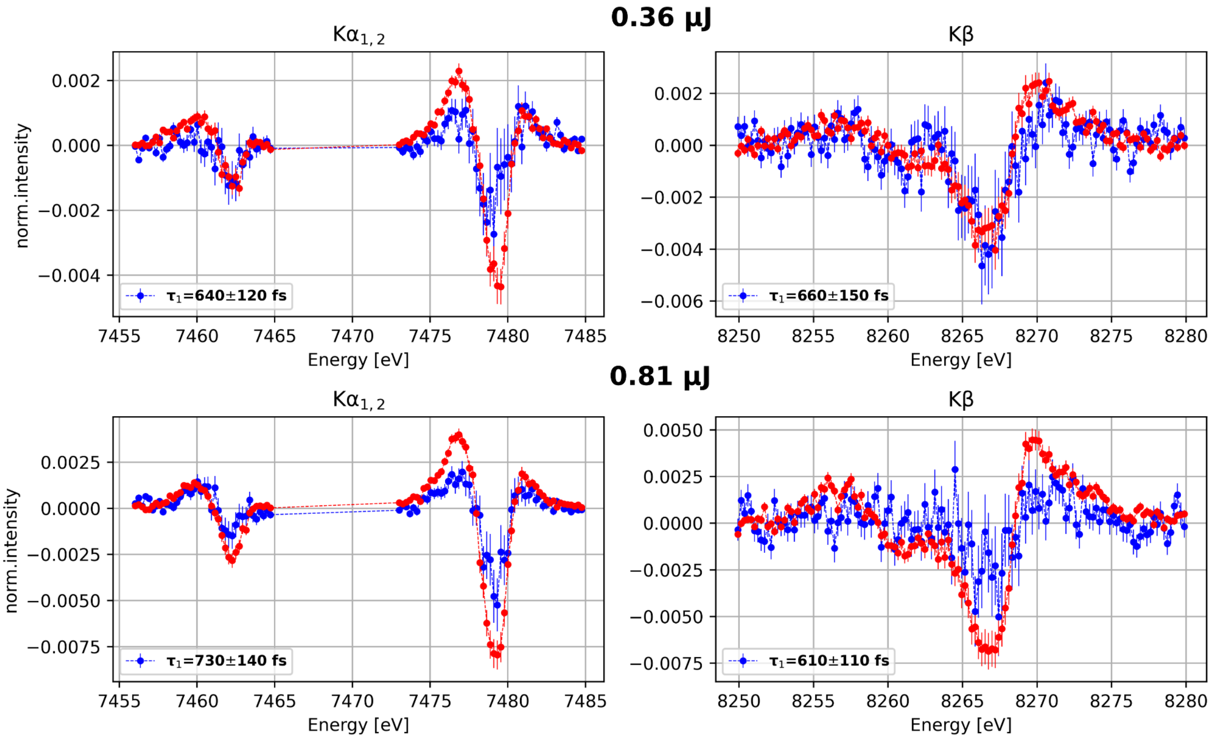

Figure S20. Global fit results for excitation with 0.36 and 0.81 μJ pump laser pulses. Left column: $K\alpha_{1,2}$; right column: Kβ. t_0 for Kβ was fixed to the best-fit value obtained in global fit of $K\alpha_1$.

Figure S21 groups the data from **Figure S22** so that the lineshapes of C1 and C2 can be compared for different powers.

Figure S22 compares the shape of component C2 and a transient lineshape taken at 50 ps time delay.

Figure S23 shows The 1D (FOM) kinetic traces and the corresponding single-exponential fits.

Table S24 and **S25** summarize all fit results, additionally reporting the results of fitting $K\alpha_1$ and $K\alpha_2$ separately. **Figure S25** shows best-fit values and confidence intervals for τ_1 for $K\alpha_1$, $K\alpha_2$, $K\alpha_{1,2}$ and Kβ datasets for 0.36 and 0.81 μJ laser excitation.

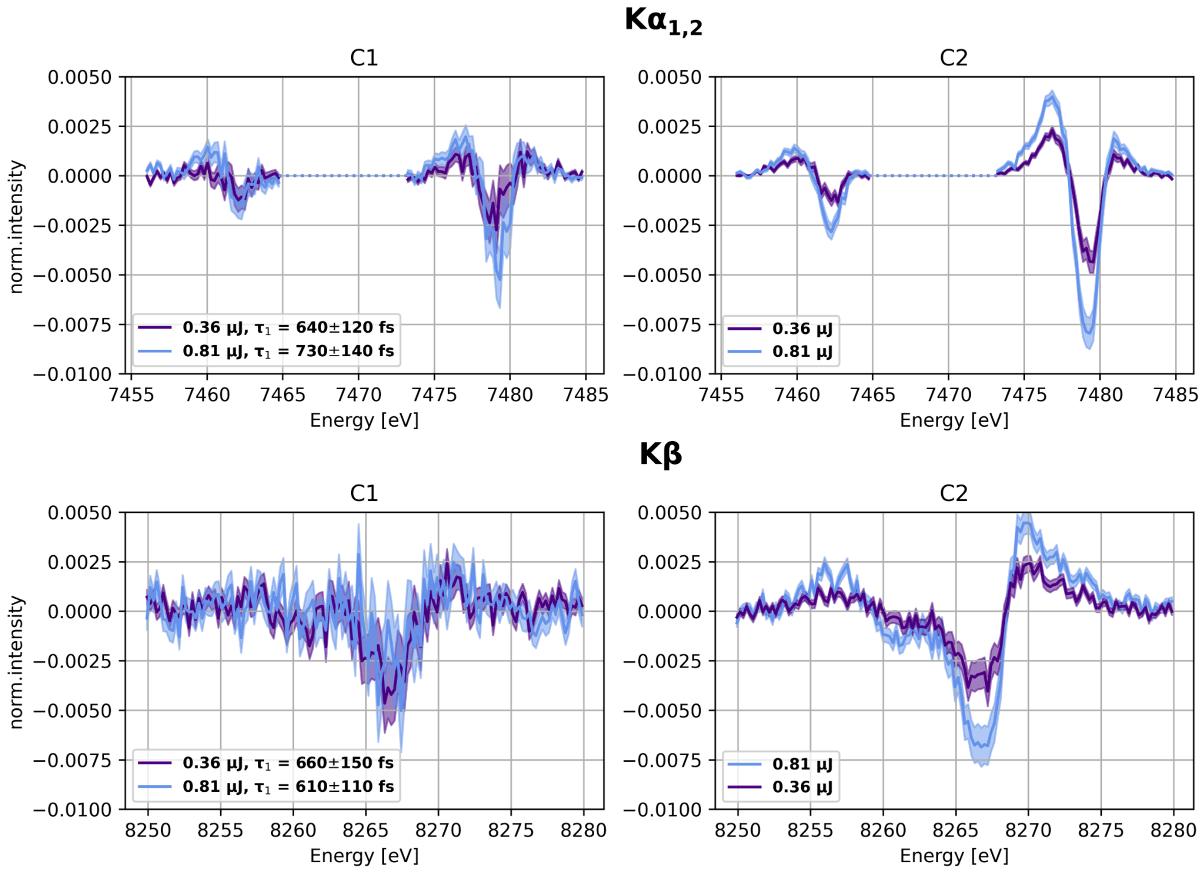

Figure S21. Lineshapes of C1 and C2 for different emission lines and pump laser pulse energies. Same data as on **Figure S20**, grouped differently. Top: Kα$_{1,2}$, bottom: Kβ. Violet: 0.36 μJ, blue: 0.81 μJ. Fitted time range: -1 to 2 ps.

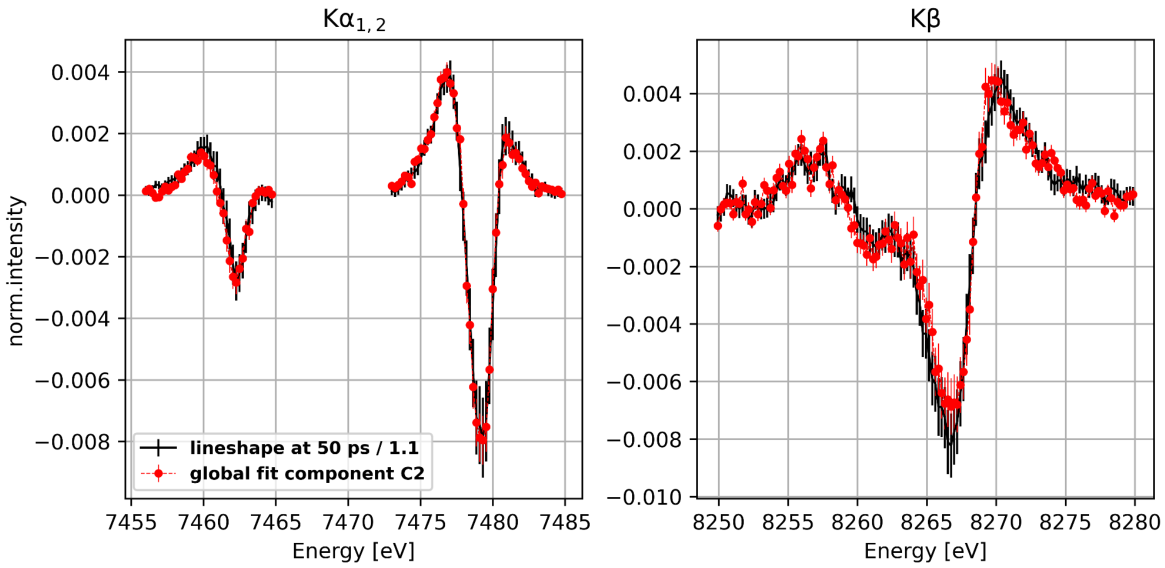

Figure S22. Comparison of C2 component and lineshape at 50 ps delay time for laser excitation with **0.81 μJ** laser pulses. The **50 ps** lineshape is divided by 1.1 to match the amplitude of C2.

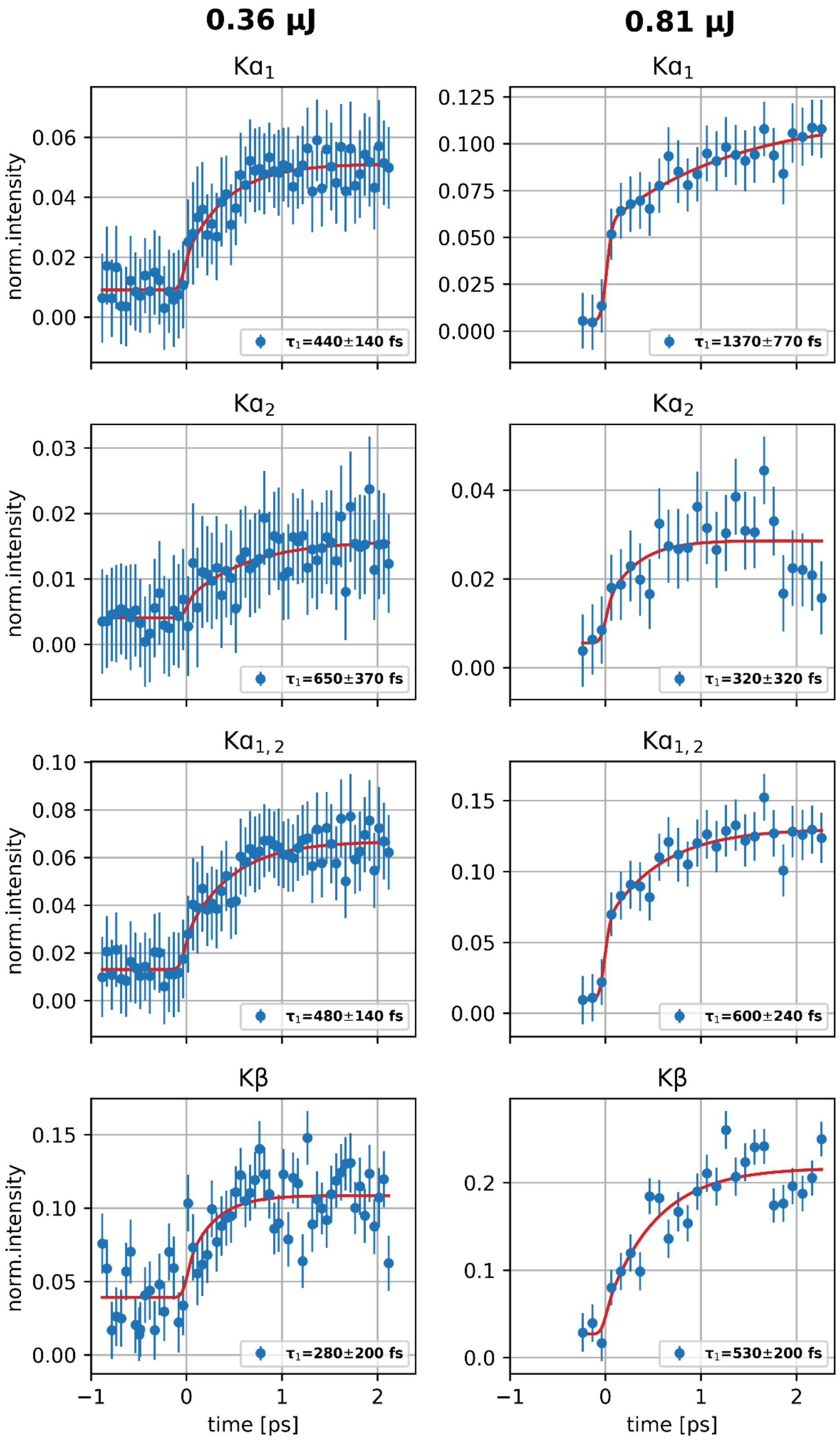

Figure S23. Short-range 1D (FOM) kinetic traces and their single-exponential fits. For Kα_1 and K$\alpha_{1,2}$ t_0 was fitted; for Ka_2 and Kβ fits are shown with t_0 being fixed to the best-fit value of global fit of the corresponding Kα_1 dataset. The fit results are summarized in tables **S27** and **S28**.

Table S24. τ_1 and t_0 best-fit values from **global fitting** of datasets for **0.36** and **0.81 µJ** pump laser pulses. t_0 was fitted for Kα_1 and K$\alpha_{1,2}$ and either fitted or fixed for Kα_2 and Kβ.

Pulse E	Kα_1	Kα_2	K$\alpha_{1,2}$	Kβ
**0.36 µJ**	τ_1 = **490** ± 100 fs<br>t_0 = ±17 fs	τ_1 = **1370** ± 800 fs<br>t_0 = 60 ± 26 fs<br>-----------------------<br>τ_1 = **1270** ± 640 fs<br>t_0 fixed	τ_1 = **640** ± 120 fs<br>t_0 = 14 ± 13 fs	τ_1 = **700** ± 160 fs<br>t_0 = -21 ± 13 fs<br>---------------------<br>τ_1 = **660** ± 150 fs<br>t_0 fixed
**0.81 µJ**	τ_1 = **870** ± 240 fs<br>t_0 = ± 16 fs	τ_1 = **540** ± 170 fs<br>t_0 = -30 ± 19 fs<br>-----------------------<br>τ_1 = **730** ± 280 fs<br>t_0 fixed	τ_1 = **730** ± 140 fs<br>t_0 = -17 ± 12 fs	τ_1 = **590** ± 100 fs<br>t_0 = -14 ± 18 fs<br>---------------------<br>τ_1 = **610** ± 110 fs<br>t_0 fixed

Table S25. Extension of **Table S24**: confidence intervals for τ_1 from **global fitting** of datasets for **0.36** and **0.81 µJ** pump laser pulses. t_0 was fitted for Kα_1 and K$\alpha_{1,2}$ and either fitted or fixed for Kα_2 and Kβ.

Pulse E	t_0	-3σ 99.73% / fs	-2σ 95.45% / fs	-1σ 68.27% / fs	**Best fit / fs**	+1σ 68.27% / fs	+2σ 95.45% / fs	+3σ 99.73% / fs
**Kα_1 (7473 – 7485 eV)**								
**0.36 µJ**	Fitted, ± 17 fs	280	330	400	**490**	630	830	1250
**0.81 µJ**	Fitted, ± 16 fs	270	350	590	**860**	1360	2800	inf
**Kα_2 (7456 – 7465 eV)**								
**0.36 µJ**	Fixed to Kα_1 value	360	510	760	**1270**	3210	inf	inf

**0.36 µJ**	Fitted, 60 ± 26 fs	350	520	780	**1370**	4350	inf	inf
**0.81 µJ**	Fixed to Kα_1 value	200	280	420	**730**	inf	inf	inf
**0.81 µJ**	Fitted, -30 ± 19 fs	160	240	350	**540**	1570	inf	inf
**K$\alpha_{1,2}$ (7456 – 7465, 7473 – 7485 eV)**								
**0.36 µJ**	Fitted, 14 ± 13 fs	350	420	510	**640**	820	1160	1920
**0.81 µJ**	Fitted, -16 ± 12 fs	330	430	550	**730**	1120	2280	inf
**Kβ (8250 – 8280 eV)**								
**0.36 µJ**	Fixed to Kα_1 value	300	380	490	**660**	960	1720	10800
**0.36 µJ**	Fitted, -21 ± 13 fs	320	410	520	**700**	1030	1860	12760
**0.81 µJ**	Fixed to Kα_1 value	330	400	490	**610**	790	1080	1650
**0.81 µJ**	Fitted, -14 ± 18 fs	320	390	480	**590**	750	1010	1540

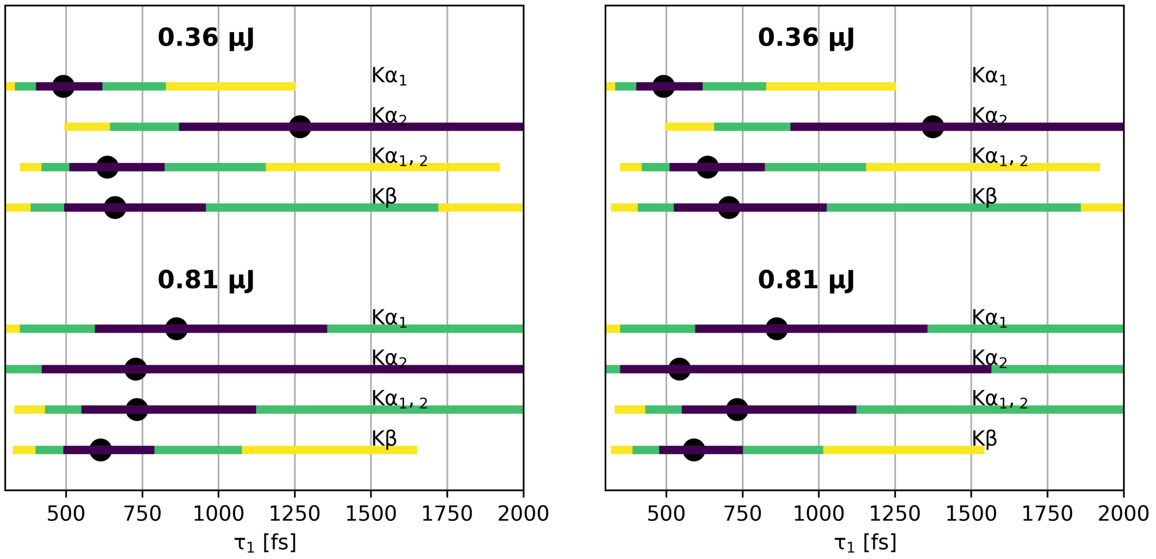

Figure S26. Graphical illustration of **Table S24** and **Table S25**: confidence intervals for fitted value of $τ_1$ for the $Kα_1$, $Kα_2$, $Kα_{1,2}$ and Kβ datasets with different powers. Violet, green and yellow lines show 1σ, 2σ and 3σ confidence intervals. Black marker shows the best-fit value. For each power, $Kα_1$, $Kα_2$ and Kβ spectra were measured simultaneously. Left figure shows results for the case when t_0 for $Kα_2$ and Kβ was fixed to best-fit value for $Kα_1$; right figure - for the case when t_0 for $Kα_2$ and Kβ was fitted freely. Results for $Kα_1$ and $Kα_{1,2}$ are the same on both figures, t_0 is fitted.

Table S27. $τ_1$ and t_0 best-fit values from **1D (FOM) fitting** of datasets for **0.36** and **0.81 μJ** pump laser pulses. t_0 was fitted for $Kα_1$ and $Kα_{1,2}$ and either fitted or fixed for $Kα_2$ and Kβ. Data and fits are shown on **Figure S23**.

**Pulse E**	**$Kα_1$**	**$Kα_2$**	**$Kα_{1,2}$**	**Kβ**
**0.36 μJ**	$τ_1$ = **440** ± 140 fs<br>t_0 =-29 ± 85 fs	$τ_1$ = **660** ± 450 fs<br>t_0 = 9 ± 130 fs<br>------------------------<br>$τ_1$ = **650** ± 370 fs<br>t_0 fixed	$τ_1$ = **480** ± 140 fs<br>t_0 = -22 ± 75 fs	$τ_1$ = **270** ± 150 fs<br>t_0 = -100 ± 300 fs<br>----------------------<br>$τ_1$ = **280** ± 200 fs<br>t_0 fixed

**0.81 µJ**	τ_1 =**1370** ± 770 fs<br><br>t_0 = 13 ± 21 fs	τ_1 = **320** ± 320 fs<br><br>t_0 fixed	τ_1 = **600** ± 240 fs<br><br>t_0 = -9 ± 40 fs	τ_1 = **540** ± 2 60 fs<br><br>t_0 = -20 ±1 70 fs<br>----------------------<br>τ_1 = **53 0** ± 200 fs<br><br>t_0 fixed

Table S28. Extension of **Table S27**: confidence intervals for τ_1 from **1D (FOM) fitting** of datasets for **0.36** and **0.81 µJ** pump laser pulses. t_0 was fitted for Kα_1 and K$\alpha_{1,2}$ and either fitted or fixed for Kα_2 and Kβ.

**Pulse E**	t_0	-3σ 99.73% / fs	-2σ 95.45% / fs	-1σ 68.27% / fs	**Best fit / fs**	+1σ 68.27% / fs	+2σ 95.45% / fs	+3σ 99.73% / fs
**Kα_1 (7474 – 7483 eV)**								
**0.36 µJ**	Fitted,<br>-29 ± 85 fs	230	280	330	**440**	600	860	1590
**0.81 µJ**	Fitted,<br>13 ± 21 fs	420	600	850	**1370**	3130	inf	inf
**Kα_2 (7456 – 7465 eV)**								
**0.36 µJ**	Fixed<br>to Kα_1 value	160	270	410	**650**	1260	26000	inf
**0.36 µJ**	Fitted,<br>9 ± 130 fs	150	270	400	**660**	1490	inf	inf
**0.81 µJ**	Fixed<br>to Kα_1 value	< 0	< 0	150	**320**	630	inf	inf
**0.81 µJ**	Fitted	No meaningful result						

**$K\alpha_{1,2}$ (7456 – 7465, 7474 – 7483 eV)**								
**0.36 µJ**	Fitted, -22 ± 75 fs	260	310	370	**480**	630	900	1640
**0.81 µJ**	Fitted, -9 ± 40 fs	230	330	440	**600**	890	1750	inf
**$K\beta$ (8253 – 8276 eV)**								
**0.36 µJ**	Fixed to $K\alpha_1$ value	<0	<0	<0	**290**	510	2040	inf
**0.36 µJ**	Fitted, -100 ± 300 fs	<0	110	180	**270**	620	inf	inf
**0.81 µJ**	Fixed to $K\alpha_1$ value	170	270	380	**530**	790	1500	inf
**0.81 µJ**	Fitted, 20 ± 170 fs	130	260	370	**540**	870	1640	inf

Conclusions:

1) Datasets at both laser pump energies show comparable value of lifetime τ_1. There is no clear dependence of τ_1 from laser pulse energy observed with the available data quality.

2) Confidence intervals for τ_1 are not symmetric: they are smaller towards short times and larger towards long times, in some cases going to infinity.

3) Data for $K\alpha_2$ and $K\beta$ are more noisy than for $K\alpha_1$. As a result, error bars / confidence intervals for $K\alpha_2$ and $K\beta$ are typically bigger.

4) Free fit of t_0 for $K\alpha_2$ and $K\beta$ sometimes results in unrealistic shift of t_0 compared to $K\alpha_1$ (likely due to high noise level); however, fixing t_0 to the value obtained in the fit of $K\alpha_1$ does not have a strong influence on the best-fit value of τ_1 for $K\alpha_2$ and $K\beta$.

5) Global-fitting 2D datasets results in smaller confidence intervals for τ_1 than fitting 1D FOM kinetic traces. The time constants are similar for both.

S.I.7.3 Species concentration

Figure S29 shows the species concentration extracted from the global-fit of $K\alpha_{1,2}$ transient signal given in **Table S24.**

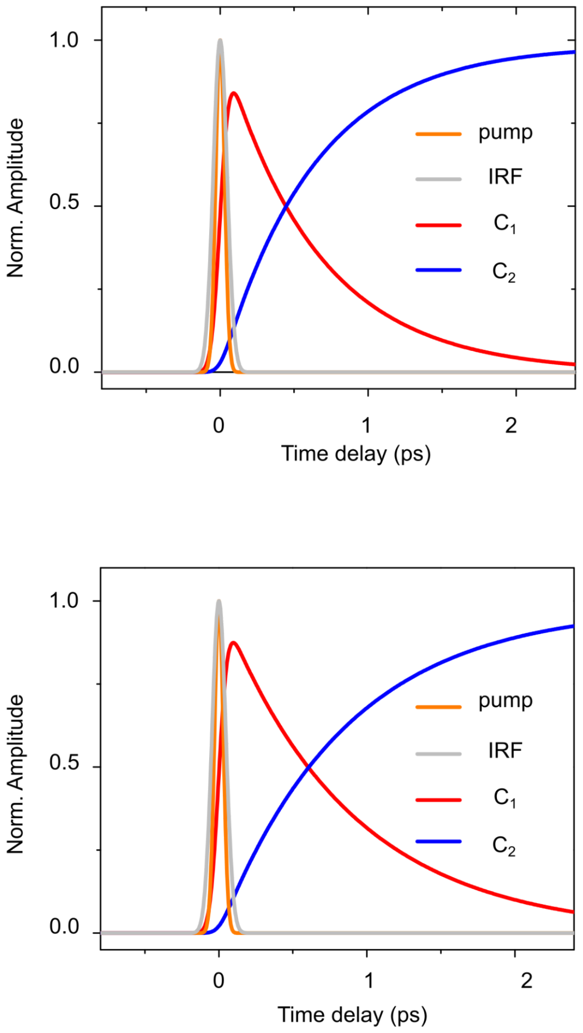

Figure S29. Species concentrations C1, C2, with IRF and pump pulse profiles obtained from the fit parameters of $K\alpha_{1,2}$ for low power (top) and high power (bottom).

SI8. Observation of CT states in low-Z transition metal complexes

The last decade has witnessed a surge in the production of novel photoactive molecular complexes based on low-Z transition metal ions exhibiting CT states that impart emissive properties and unconventional photoreactivity. Systematic studies combining innovative synthesis [1], ultrafast spectroscopic techniques from the IR to the X-ray [2, 3] and advanced theoretical modeling [4] have enabled large strides in the elaboration of structure-properties relationships in the family of octahedral d^6 complexes.

Similar efforts are now taking place for the family of square-planar d^8 complexes. **Table S30** summarizes the Ni(II) complexes for which the deactivation pathways have been studied, including the characters and the lifetimes of the intermediate and metastable states, when reported. The numbers attributed to the complexes refer to the ones used in the respective publications. Close inspection confirms the influence of the coordination and external environment (solution vs solid). General trends have been delineated. [1, 5, 6] Future systematic studies are necessary to further benchmark their applicability.

Table S30. Table summarizing known square planar Ni(II) complexes, their physical state (solid or solution with the solvent given in para,thesis), the excitation wavelength (nm) and the deactivation pathways.

	**State**	**Exc (nm)**	**Deactivation**
**Cope *et al* (7)**			
**1**	solid	405	triplet emission
**2**	solid	405	triplet emission
**3**	solid	405	triplet emission
**4**	solid	405	triplet emission
**5**	solid	405	triplet emission
**Shields *et al* (8)**			
**1-Cl**	Solution (THF)	295	^{3}MLCT : 2.3 ps^{-1} , ^{3}dd : 5.5×10^{-4} ps^{-1}
**1-Cl**	Solution (THF)	400	^{3}MLCT : 0.86 ps^{-1} , ^{3}dd : 2.3×10^{-4} ps^{-1}
**1-Br**	Solution (THF)	295	^{3}MLCT : 2.6, ps^{-1} , ^{3}dd : 1.3×10^{-4} ps^{-1}
**1-Br**	Solution (THF)	400	^{3}MLCT : 0.72ps^{-1}, ^{3}dd : 1.1×10^{-4} ps^{-1}
**1-I**	Solution (THF)	295	^{3}MLCT : 3.2 ps^{-1} , ^{3}dd : 1.6×10^{-3} ps^{-1}

**1-I**	Solution (THF)	400	^{3}MLCT : 0.11 ps^{-1} , ^{3}dd : 5.5×10^{-6} ps^{-1}
**Wong *et al* (9)**			
**1**	solution/ solid, RT/low-T	365	non emissive
**2**	solution (CH_2Cl_2, RT)	365	non emissive
**2**	solution (CH_2Cl_2, low-T)	365	triplet emission (0.43 μs)
**2**	solid (RT)	365	triplet emission (N/A)
**2**	solid (low-T)	365	triplet emission (0.11 μs)
**3**	solution/ solid, RT/low-T	365	non emissive
**Ting *et al* (10)**			
**1^{OMe}**	solution (THF)	530	^{3}MLCT : (0.69 ps, 4.77 ps), ^{3}dd : 2.65 ns
**1^{OMe}**	solution (benzene)	530	^{3}MLCT : (0.82 ps, 6.91 ps), ^{3}dd : 2.97 ns
**1^{t-Bu}**	solution (THF)	550	^{3}MLCT : (0.66 ps, 6.89 ps), ^{3}dd : 4.00 ns
**1^{t-Bu}**	solution (benzene)	550	^{3}MLCT : (1.18 ps, 12.8 ps), ^{3}dd : 3.95 ns
**1^{H}**	solution (THF)	530	^{3}MLCT :(0.62 ps, 5.45 ps), ^{3}dd :4 .13 ns
**1^{H}**	solution (benzene)	530	^{3}MLCT : (0.83 ps, 12.8 ps), ^{3}dd : 4.19 ns
**1^{Ph}**	solution (THF)	570	^{3}MLCT : (0.70 ps, 7.77 ps), ^{3}dd : 3.77 ns
**1^{Ph}**	solution (benzene)	570	^{3}MLCT : (0.99 ps, 13.6 ps), ^{3}dd : 3.88 ns
**1^{CO2Et}**	solution (THF)	590	^{3}MLCT : (0.81 ps, 8.31 ps), ^{3}dd : 4.44 ns
**1^{CO2Et}**	solution (benzene)	590	^{3}MLCT : (0.73 ps, 7.89 ps), ^{3}dd : 4.64 ns
**2^{OMe}**	solution (THF)	550	^{3}MLCT : (0.98 ps, 14.3 ps), ^{3}dd : 8.10 ns
**2^{OMe}**	solution (benzene)	550	^{3}MLCT : (1.22 ps, 13.7 ps), ^{3}dd : 7.50 ns
**2^{H}**	solution (THF)	550	^{3}MLCT : (0.69 ps, 5.58 ps), ^{3}dd : 6.04 ns
**2^{H}**	solution (benzene)	550	^{3}MLCT : (1.03 ps, 10.3 ps), ^{3}dd : 6.35 ns
**2^{CF3}**	solution (THF)	510	^{3}MLCT : (1.03 ps, 7.69 ps), ^{3}dd : 6.75 ns
**2^{CF3}**	solution (benzene)	510	^{3}MLCT : (1.23 ps, 11.7 ps), ^{3}dd : 5.98 ns

**Kurz *et al* (11)**			
**1**	solution ($CHCl_3$)	385	triplet emission<br>ligand based
**2**	solution ($CHCl_3$)	385	triplet emission<br>ligand based
**3**	solution ($CHCl_3$)	385	triplet emission<br>ligand based
**Ogawa *et al* (5)**			
**1**	solution (CH_3CN)	380	non emissive<br>^{3}MLCT : 7.3 ps, ^{3}dd : 133 ps
**1**	solution (CH_2Cl_2)	380	non emissive<br>^{3}MLCT : 48 ps, ^{3}dd : 14 ps
**2**	solution (CH_3CN)	380	non emissive<br>^{3}MLCT : 5.8 ps, ^{3}dd : 40 ps
**2**	solution (CH_2Cl_2)	380	non emissive<br>^{3}MLCT : 17 ps, ^{3}dd : 49 ps
**3**	solution (CH_3CN)	380	non emissive<br>^{3}MLCT : 0.6 ps, ^{3}dd : 24 ps
**3**	solution (CH_2Cl_2)	380	non emissive<br>^{3}MLCT : 0.5 ps, ^{3}dd : 38 ps
**Ogawa *et al* (6)**			
**1**	solution (CH_3CN)	400	non emissive<br>^{3}MLCT : 0.15 ps, ^{3}dd : 14 ps
**1**	solution (CH_2Cl_2)	400	non emissive<br>^{3}MLCT : 0.3 ps, ^{3}dd : 21 ps
**2**	solution (CH_3CN)	400	non emissive<br>^{3}MLCT : 0.7 ps, ^{3}dd : 9.2 ps
**2**	solution (CH_2Cl_2)	400	non emissive<br>^{3}MLCT : 0.9 ps, ^{3}dd : 14 ps
**Hou *et al* (12)**			

**1a**	solid	365	triplet emission
**2a**	solid	365	triplet emission
**3a**	solid	365	triplet emission
**1b**	solid	365	triplet emission
**2b**	solid	365	triplet emission
**3b**	solid	365	triplet emission

References

(1) Sinha, N.; Wenger, O. S. Photoactive Metal-To-Ligand Charge Transfer Excited States in 3d6 Complexes with Cr^0, Mn^I, Fe^{II}, and Co^{III}. *Journal of the American Chemical Society* 2023, *145* (9), 4903–4920.

(2) Hainer, F.; Alagna, N.; A. Reddy Marri; Penfold, T. J.; Gros, P. C.; Haacke, S.; T. Buckup. Vibrational Coherence Spectroscopy Identifies Ultrafast Branching in an Iron(II) Sensitizer. *The Journal of Physical Chemistry Letters* 2021, *12* (35), 8560–8565.

(3) Gaffney, K. J. Capturing Photochemical and Photophysical Transformations in Iron Complexes with Ultrafast X-Ray Spectroscopy and Scattering. Chemical Science 2021, 12 (23), 8010–8025.

(4) Mátyás Pápai, et al. “Ultrafast 3MLCT Quenching and Vibrational Coherence: Excited-State Dynamics of the First-Discovered Fe(II)–Carbene Sensitiser Resolved.” *Journal of Materials Chemistry. A, Materials for Energy and Sustainability*, vol. 11, no. 47, 1 Jan. 2023, pp. 25955–25962.

(5) Ogawa, T.; Sinha, N.; Pfund, B.; Prescimone, A.; Wenger, O. S. Molecular Design Principles to Elongate the Metal-to-Ligand Charge Transfer Excited-State Lifetimes of Square-Planar Nickel(II) Complexes. *Journal of the American Chemical Society* 2022, *144* (48), 21948–21960.

(6) Ogawa, T.; Wenger, O. S. Nickel(II) Analogues of Phosphorescent Platinum(II) Complexes with Picosecond Excited‐State Decay. *Angewandte Chemie International Edition* **2023**, *62* (46).

(7) J. D. Cope, J. A. Denny, R. W. Lamb, L. E. McNamara, N. I. Hammer, C. E. Webster, T. K. Hollis, *J. Organomet. Chem.* 2017, *845*, 258–265.

(8) Shields, B. J. ; Kudisch, B. ; Scholes, G. D. ; Doyle, A. G. Long-Lived Charge-Transfer States of Nickel(II) Aryl Halide Complexes Facilitate Bimolecular Photoinduced Electron Transfer. *Journal of the American Chemical Society* 2018, *140* (8), 3035-3039.

(9) Wong, Y.-S.; Tang, M.-C.; Ng, M.; Vivian Wing-Wah Yam. Toward the Design of Phosphorescent Emitters of Cyclometalated Earth-Abundant Nickel(II) and Their Supramolecular Study. *Journal of the American Chemical Society* 2020, *142* (16), 7638–7646.

(10) Ting, S. I. ; Garakyaraghi, S. ; Taliaferro, C. M. ; Shields, B. J. ; Scholes, G. D. ; Castellano, F. N. ; Doyle, A. G. 3D-D Excited States of Ni(II) Complexes Relevant to Photoredox Catalysis : Spectroscopic Identification and Mechanistic Implications. *Journal of the American Chemical Society* 2020, *142* (12), 5800-5810.

(11) H. Kurz, K. Schötz, I. Papadopoulos, F. W. Heinemann, H. Maid, D. M. Guldi, A. Köhler, G. Hörner, B. Weber, *J. Am. Chem. Soc.* 2021, *143*, 3466–3480.

(12) C.-L. Hou, J.-X. Song, X. Chang, Y. Chen, Photoluminescent nickel(II) carbene complexes with ligand-to-ligand charge-transfer excited states, *Chin. Chem. Lett.* 2024, *144*, 108333.
